# Integrated respirometry and metabolomics unveil circadian metabolic dynamics in *Drosophila*

**DOI:** 10.7554/eLife.108681

**Published:** 2026-09-24

**Authors:** Farheen Akhtar, Dania M Malik, Arjun Sengupta, Paula Haynes, Andrew D Nguyen, C Jaco Klok, Amita Sehgal, Aalim Weljie

**Affiliations:** 1 Department of Systems Pharmacology and Translational Therapeutics, University of Pennsylvania Philadelphia United States; 2 Chronobiology and Sleep Institute, University of Pennsylvania Philadelphia United States; 3 Institute for Translational Medicine and Therapeutics, University of Pennsylvania Philadelphia United States; 4 Howard Hughes Medical Institute, University of Pennsylvania Philadelphia United States; 5 Department of Neuroscience, Perelman School of Medicine, University of Pennsylvania Philadelphia United States; 6 Sable Systems International North Las Vegas United States; Wake Forest University School of Medicine United States; New York University United States

**Keywords:** circadian rhythms, sleep, metabolism, respirometry, metabolomics, metabolic flexibility, *D. melanogaster*

## Abstract

Sleep and circadian rhythms shape organismal energy patterns, but how this timing connects to oxygen use and carbon dioxide production remains incompletely understood. We combined high-resolution respirometry with liquid chromatography-mass spectrometry (LC-MS)-based metabolomics to characterize respiratory dynamics and metabolic states in *Drosophila melanogaster*, resolving genotype-specific impacts of sleep disruption and circadian regulation. Wild-type flies under light-dark cycles (WT-LD) showed rhythmic respiratory patterns reflective of anticipatory coordination of mitochondrial energy metabolism, amino acid turnover, and redox cycling. Short-sleep mutants (*fmn*, *sss*) exhibited elevated metabolic rates, with reactive shifts of fuel preferences toward lipid and amino acid catabolism, and altered mitochondrial respiration. The clock mutant (*per^01^*) and flies under constant darkness (WT-DD) showed reactive and widespread metabolic dysregulation and impaired redox homeostasis. These findings demonstrate that both sleep and circadian systems contribute to aligning metabolic substrate selection with energy demands, offering mechanistic insights into how disruptions in behavioral states compromise metabolic health.

## Introduction

Metabolism is a fundamental physiological process responsible for coordinating energy production and substrate utilization in response to changing physiological demands. This process is dynamically regulated by behavioral states, such as sleep and wakefulness, as well as directly by internal circadian clocks that align physiology with the external environment (Bass and Takahashi, 2010; Panda, 2016; Sharma and Kavuru, 2010). Disruptions in these temporal processes are strongly associated with metabolic disorders, including obesity and type 2 diabetes (Aurora and Punjabi, 2013; Leproult and Van Cauter, 2010; Reutrakul and Van Cauter, 2014), as well as other metabolic diseases such as cardiovascular dysfunction and cancer (Sulli et al., 2018).

With conserved and well-characterized sleep and circadian mechanisms (Hendricks et al., 2000; Allada and Siegel, 2008), as well as metabolic pathways also found in mammals, *Drosophila* offers a genetically tractable model for dissecting interactions between these different systems. In particular, sleep mutants with robust phenotypes can be employed to determine how loss of sleep impacts metabolic parameters, including rhythms of metabolic activity. At the same time, additional tools are now available for metabolic measurements previously not possible. Multiple approaches can be combined for a comprehensive understanding of metabolism under precisely controlled environmental conditions and at high temporal resolution (Donelson et al., 2012).

Indirect calorimetry is a non-invasive form of respirometry that enables real-time quantification of whole-organism metabolic rate by measuring oxygen consumption (VO_2_) and carbon dioxide production (VCO_2_) (Speakman, 2013; Even et al., 1994). These measurements allow for the estimation of energy expenditure and respiratory quotient (RQ), which can provide an index of relative substrate utilization, with appropriate caveats (McClave and Snider, 1992; Frayn, 1983). Although widely applied in mammalian systems (Arch et al., 2006), indirect calorimetry in small model organisms such as *Drosophila melanogaster* has only recently become feasible with advances in high-resolution, small-volume flow-through systems (Yatsenko et al., 2014; Wiggin et al., 2020; Brown et al., 2022). This is a critical advancement, as dissecting time-of-day-dependent metabolic regulation in mammals is often confounded by overlapping effects of sleep, activity, and feeding rhythms (Cedernaes et al., 2015; Katayose et al., 2009). While this can also be true in flies, the genetic tools available allow distinction between these processes to a greater extent.

This study focuses on wild-type (WT) isogenic control flies alongside three genetically characterized mutants, including two short-sleep mutants: *fumin* (*fmn*), which displays impaired dopamine transporter function and defective dopamine reuptake (Kume et al., 2005), and *sleepless* (*sss*), which lacks the Sleepless protein, resulting in altered membrane excitability and reduced GABA signaling (Koh et al., 2008; Chen et al., 2015; Wu et al., 2010); and the circadian mutant *period^01^* (*per^01^*), which lacks a functional molecular clock (Konopka and Benzer, 1971). To investigate how sleep and circadian disruption independently and interactively influence metabolic regulation, we utilized a small-animal respirometry platform built on the MAVEn-FT system, coupled with a Licor 7000 CO_2_ analyzer and an Oxzilla differential O_2_ analyzer. This system builds upon the sleep and activity metabolic monitor, which first demonstrated the feasibility of simultaneous CO_2_ and O_2_ measurements in *Drosophila* (Stahl et al., 2017). This MAVEn-based platform enhances temporal resolution and throughput, enabling continuous measurement of respiratory parameters across multiple groups of flies over daily cycles, and allowing fine-scale profiling of metabolic rhythms and their disruption across sleep-wake cycles. In this study, we interpret RQ conservatively as reflecting relative shifts in substrate utilization over time and between genotypes, rather than as a precise quantitative measure of absolute carbohydrate versus lipid oxidation.

To provide a more comprehensive perspective on metabolic regulation, we complemented newly generated respiratory measurements with steady-state metabolomic profiling using liquid chromatography-mass spectrometry (LC-MS) data previously published from our group (Malik et al., 2024). This integrative framework enabled time-matched and lag-aware analysis of respiratory output and metabolite profiles across Zeitgeber time (ZT) in the LD cycle, providing detailed insights into how sleep and circadian mutations influence metabolic homeostasis (Malik et al., 2024; Yuan et al., 2012; Rhoades et al., 2018). By employing this framework, we sought to uncover conserved mechanisms linking temporal biological processes, such as sleep and circadian rhythms, to metabolic homeostasis and to elucidate how genetic and environmental perturbations in these systems influence energy balance and metabolic regulation.

## Results

### Metabolic fuel utilization across genotypes based on RQ

**Figure 1.**
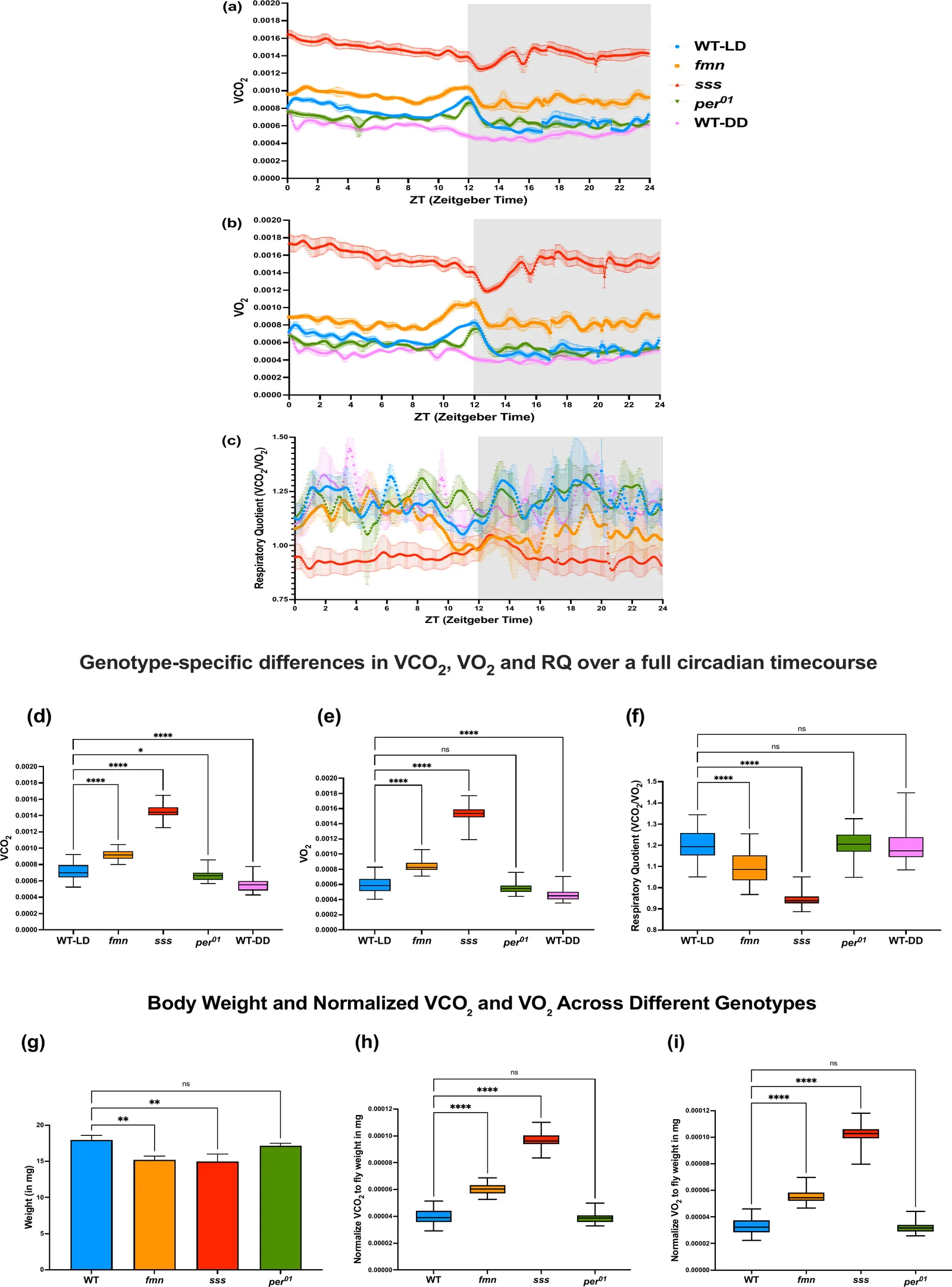
VCO2, VO2 and RQ profiles across teh circadian cycle in different genotypes. (**a–c**) VCO_2_, VO_2_, and respiratory quotient (RQ) profiles across the 24 hr cycle in different genotypes. VCO_2_, VO_2_, and RQ (VCO_2_/VO_2_) were continuously recorded at 1 s intervals from Zeitgeber time (ZT) 0–24 and subsequently averaged into 5 min bins for analysis. Traces represent mean VCO_2_, VO_2_, and RQ values across the 24 hr recording period for wild-type flies under light-dark conditions (WT-LD), short-sleep mutants *fumin* (*fmn*) and *sleepless* (*sss*), the circadian clock mutant *period*^01^ (*per*^01^), and wild-type flies maintained in constant darkness (WT-DD). Source data are the continuous respirometry recordings. (**d–f**) Genotype-specific differences in VCO_2_, VO_2_, and RQ measured over a full 24 hr recording period. Boxplots show average values of (left to right) RQ, carbon dioxide production (VCO_2_), and oxygen consumption (VO_2_) across genotypes and lighting conditions. Measurements were taken continuously over a 24 hr period using a flow-through MAVEn system. Genotypes include WT-LD and WT-DD, short-sleep mutants *fumin* (*fmn*) and *sleepless* (*sss*), and circadian mutant *per^01^*. Group differences were assessed using the Kruskal-Wallis test, followed by Dunn’s multiple comparisons post hoc test. Significance is denoted as: p<0.05 (*)*,* p<0.01 (**), p<0.001 (***); ns = not significant. (**g–i**) Body weight and normalized VCO_2_ and VO_2_ across different genotypes. Body weight (mg) and respiratory parameters (VCO_2_ and VO_2_) normalized to body weight (mg) were measured in wild-type (WT), *fmn*, *sss*, and *per^01^* mutant flies. Statistical significance was assessed using one-way ANOVA followed by Dunnett’s multiple comparisons test against WT. p<0.05 (*), p<0.01 (**), p<0.001 (***); ns = not significant. For all panels with error bars or shaded error bands, values are presented as mean ± SEM. Data represent approximately 300 flies per genotype across 3 experimental days (25 flies/chamber, 4 chambers/experiment). The chamber was used as the experimental unit; *n* denotes the number of chambers.

To assess whole-body metabolic activity, we performed respirometry on groups of 25 flies per genotype, measuring oxygen consumption (VO_2_) and carbon dioxide production (VCO_2_) across the 24 hr cycle. The RQ, calculated as the ratio of VCO_2_ to VO_2_, provides insight into the predominant metabolic substrate being utilized: an RQ of 1.0 reflects pure carbohydrate metabolism, values below 1.0 indicate lipid and protein oxidation, and values above 1.0 suggest lipogenesis (the conversion of carbohydrates into fats) (Frayn, 1983). RQ, VCO_2_, and VO_2_ were continuously recorded from ZT 0–24 at 1 s intervals and subsequently averaged into 5 min bins for analysis. Figure 1a–c shows the 5 min binned VCO_2_, VO_2_, and RQ profiles across the 24 hr cycle for all genotypes (separate individual plots for VCO_2_, VO_2_, and RQ for each genotype are provided in Figure 1—figure supplements 1a–e, 2a–e, 3a–e, respectively; replicate information for respirometry is provided in Supplementary file 1C). The average values of VCO_2_, VO_2_, and RQ across the full 24 hr cycle are summarized in Figure 1d–f.

**Figure 1—figure supplement 1.**
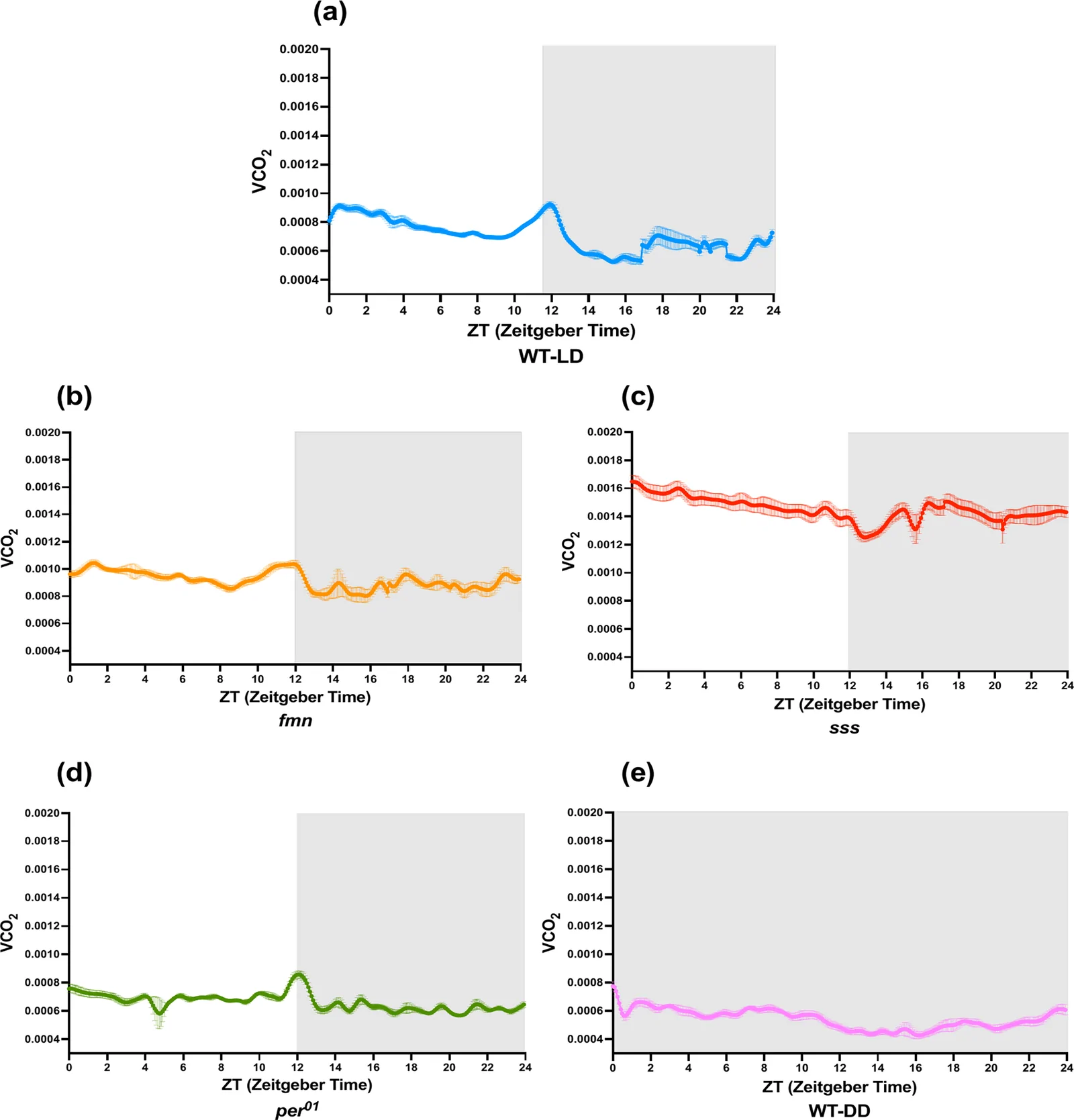
VCO_2_ profiles across the 24 hr recording period in different genotypes. (**a–e**) VCO_2_ was continuously measured at 1 s intervals from Zeitgeber time (ZT) 0–24 and averaged into 5 min bins for analysis. The traces display mean VCO_2_ values across the 24 hr recording period for the following genotypes: wild-type flies under light-dark conditions (WT-LD), short-sleep mutants *fumin* (*fmn*) and *sleepless* (*sss*), the circadian clock mutant *period*^01^ (*per^01^*), and wild-type flies in constant darkness (WT-DD). For all panels, traces are presented as mean ± SEM. Data represent approximately 300 flies per genotype across 3 experimental days (25 flies/chamber, 4 chambers/experiment). The chamber was used as the experimental unit; *n* denotes the number of chambers.

**Figure 1—figure supplement 2.**
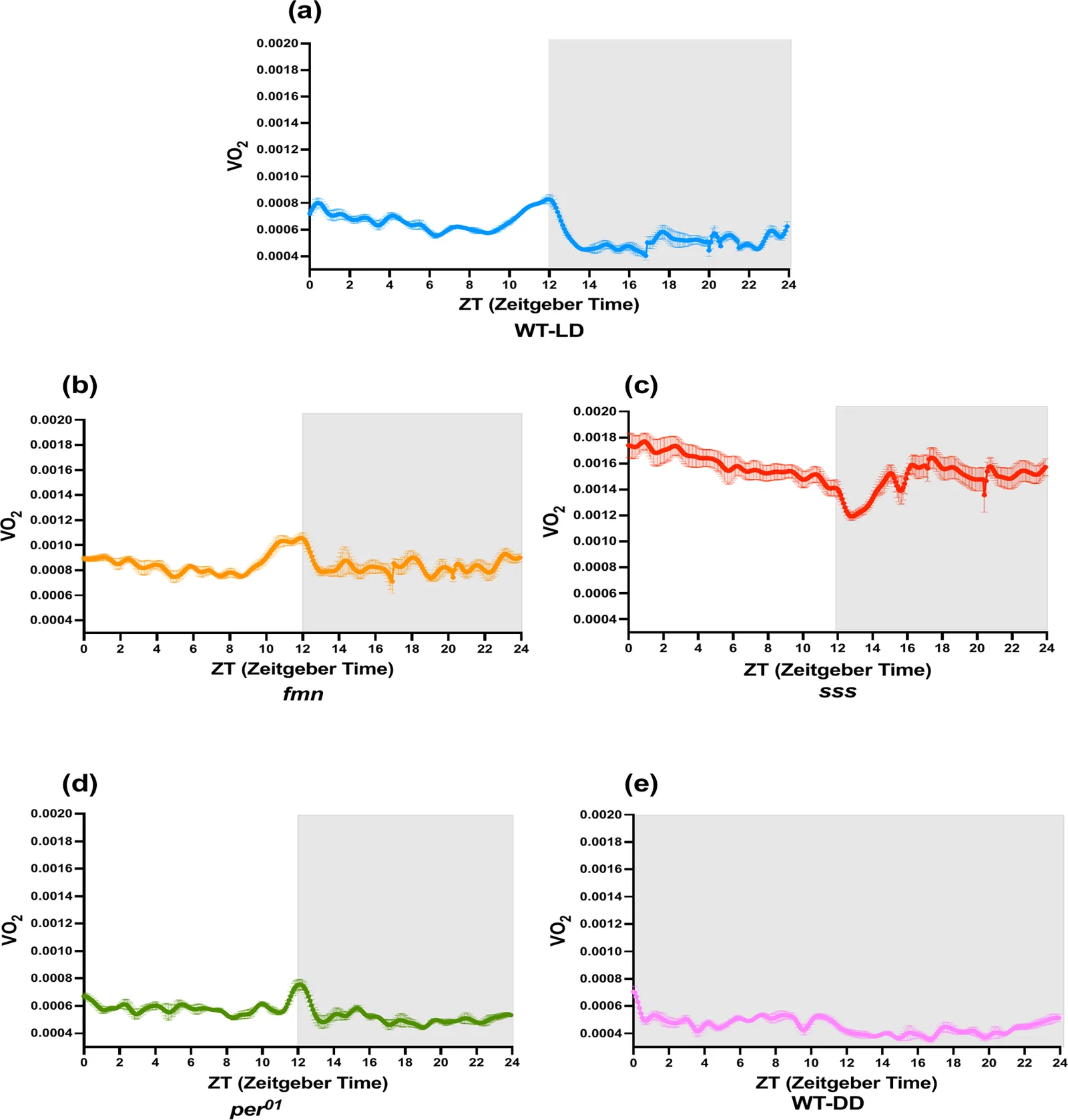
VO_2_ profiles across the 24 hr recording period in different genotypes. (**a–e**) VO_2_ was continuously measured at 1 s intervals from Zeitgeber time (ZT) 0–24 and averaged into 5 min bins for analysis. The traces display mean VO_2_ values across the 24 hr recording period for the following genotypes: wild-type flies under light-dark conditions (WT-LD), short-sleep mutants *fumin* (*fmn*) and *sleepless* (*sss*), the circadian clock mutant *period*^01^ (*per^01^*), and wild-type flies in constant darkness (WT-DD). For all panels, traces are presented as mean ± SEM. Data represent approximately 300 flies per genotype across 3 experimental days (25 flies/chamber, 4 chambers/experiment). The chamber was used as the experimental unit; *n* denotes the number of chambers.

**Figure 1—figure supplement 3.**
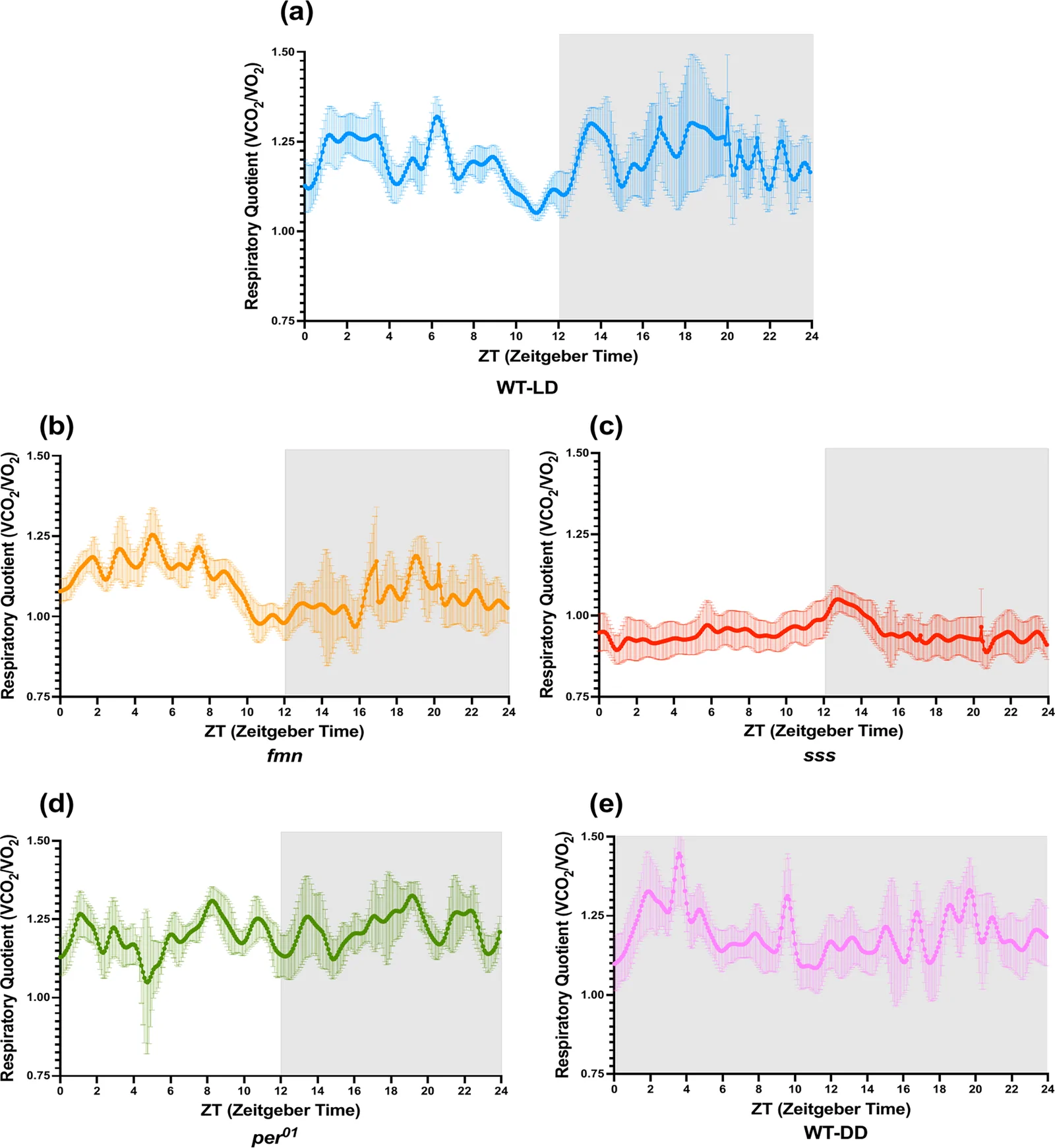
Respiratory quotient (RQ) profiles across the 24 hr recording period in different genotypes. (**a–e**) RQ (VCO_2_/VO_2_) was continuously recorded at 1 s intervals from Zeitgeber time (ZT) 0–24 and subsequently averaged into 5 min bins for analysis. Traces represent mean RQ values across the 24 hr recording period for wild-type flies under light-dark conditions (WT-LD), short-sleep mutants *fumin* (*fmn*) and *sleepless* (*sss*), the circadian clock mutant *period*^01^ (*per^01^*), and wild-type flies maintained in constant darkness (WT-DD). For all panels, traces are presented as mean ± SEM. Data represent approximately 300 flies per genotype across 3 experimental days (25 flies/chamber, 4 chambers/experiment). The chamber was used as the experimental unit; *n* denotes the number of chambers.

**Figure 1—figure supplement 4.**
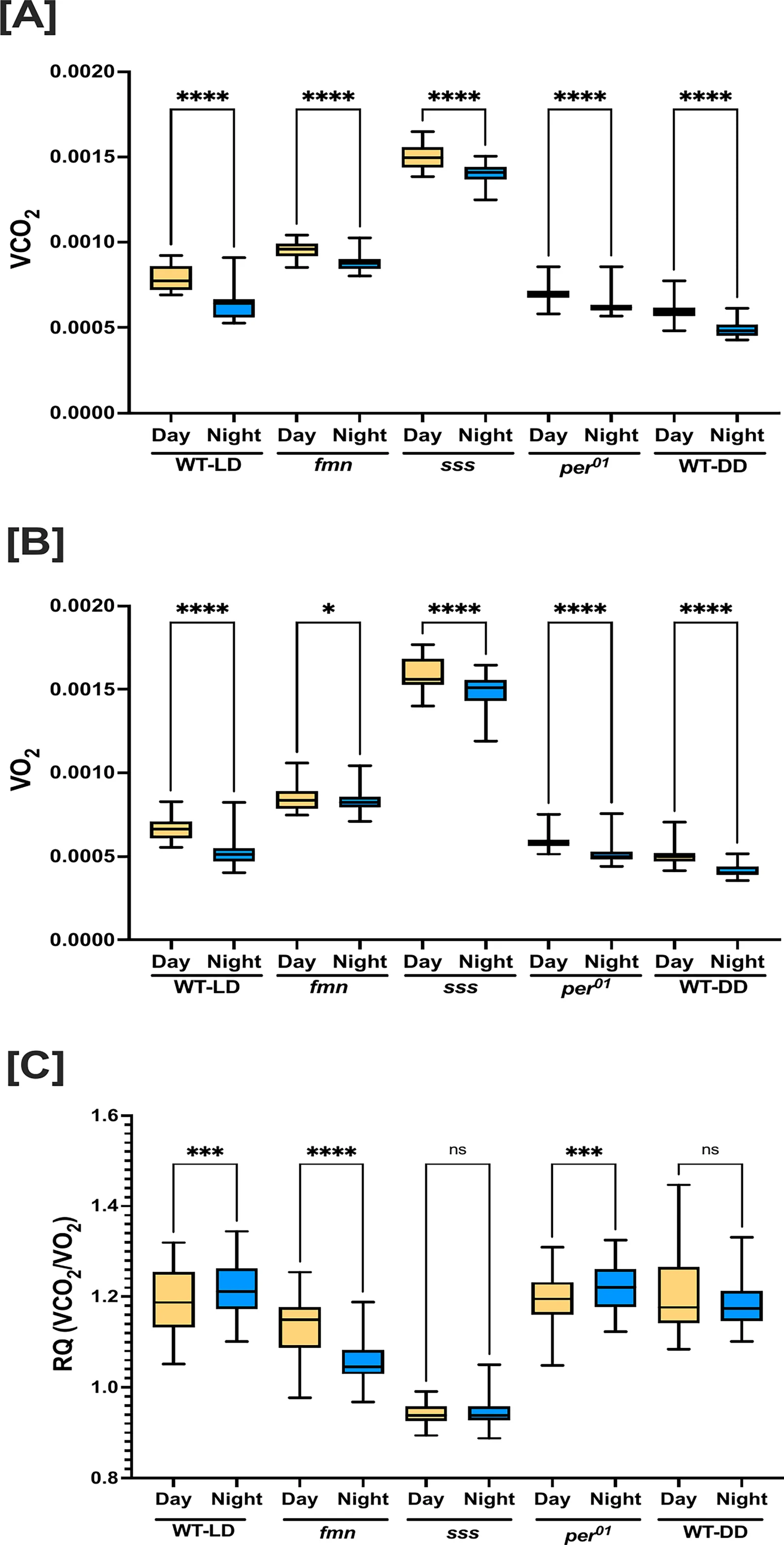
Day-night differences in genotype-specific metabolic rates. Boxplots depict day (Zeitgeber time [Z]T0–12) and night (ZT12–24) averages plotted side-by-side for each genotype to facilitate direct day-night comparison. Panels show genotype-specific differences in (**A**) carbon dioxide production (VCO_2_), (**B**) oxygen consumption (VO_2_), and (**C**) respiratory quotient (RQ). Genotypes include wild-type flies under light-dark conditions (WT-LD), wild-type flies maintained in constant darkness (WT-DD), short-sleep mutants *fumin* (*fmn*) and *sleepless* (*sss*), and the circadian mutant *period^01^* (*per^01^*). Measurements were acquired continuously using a flow-through MAVEn system and binned into day and night intervals. Group differences were assessed using the Kruskal-Wallis test followed by Dunn’s multiple comparisons post hoc test. Significance is denoted as: p<0.05 (*), p<0.01 (**), p<0.001 (***); ns = not significant. Source data are the same continuous respirometry recordings shown in Figure 1. Data represent approximately 300 flies per genotype across 3 experimental days (25 flies/chamber, 4 chambers/experiment). The chamber was used as the experimental unit; *n* denotes the number of chambers.

Among the genotypes tested, WT-LD, WT-DD, and *per^01^* flies exhibited similarly elevated RQ values (1.20, 1.19, and 1.20, respectively), consistent with active lipogenesis. Group differences were assessed using the Kruskal-Wallis test, followed by Dunn’s multiple comparisons post hoc test, which revealed no statistically significant differences in RQ among these three genotypes (Figure 1f, ns), suggesting that neither free-running conditions in constant darkness (WT-DD) nor loss of clock function in the *per^01^* mutant alters the dominant fuel utilization strategy under basal conditions.

In contrast, short-sleeping mutants displayed lower RQ values. The *fmn* mutant exhibited a moderate reduction (RQ = 1.09), reflecting increased reliance on carbohydrate metabolism. The *sss* mutant showed the lowest RQ (0.94), indicating a shift toward lipid and protein catabolism. Both mutants were significantly different from WT-LD (p<0.001), highlighting genotype-specific alterations in fuel utilization associated with sleep loss (Figure 1f).

These results demonstrate that WT-LD, WT-DD, and *per^01^* flies maintain a lipogenic profile, whereas sleep-deficient mutants exhibit altered substrate utilization, favoring catabolism of carbohydrates or lipids depending on the severity of sleep disruption.

**Figure 2.**
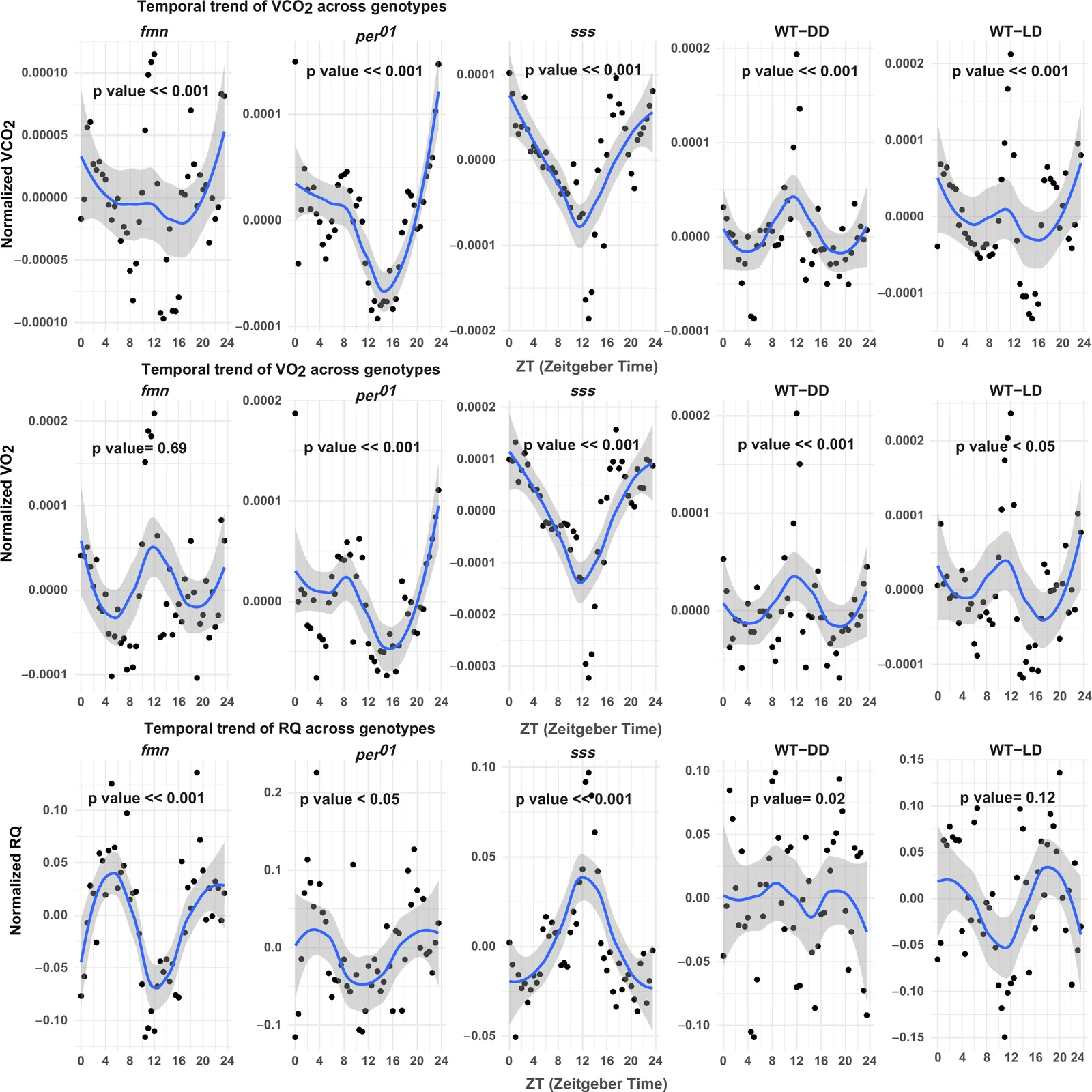
Twenty-four-hr time course of respiratory parameters across genotypes. Normalized temporal profiles of carbon dioxide production (VCO_2_) (top), oxygen consumption (VO_2_) (middle), and respiratory quotient (RQ) (bottom) plotted over a 24 hr cycle (Zeitgeber time [Z]T0–24) for genotypes: wild-type in light-dark (WT-LD) and constant darkness (WT-DD), short-sleep mutants *fumin* (*fmn*) and *sleepless* (*sss*), and circadian mutant *per^01^*. Curves reveal genotype-specific rhythmicity and metabolic dynamics across the 24 hr recording period. Significant genotype×time interactions were observed for all variables (VCO_2_, VO_2_, RQ), indicating genotype-dependent alterations in respiratory rhythmicity. p<0.05 considered significant. For all panels with error bars or shaded error bands, values are presented as mean ± SEM. Source data are the same continuous respirometry recordings shown in Figure 1; Figure 2 presents rhythmicity analysis of VCO_2_, VO_2_, and RQ across the 24 hr cycle. Data represent approximately 300 flies per genotype across 3 experimental days (25 flies/chamber, 4 chambers/experiment). The chamber was used as the experimental unit; *n* denotes the number of chambers.

**Figure 3.**
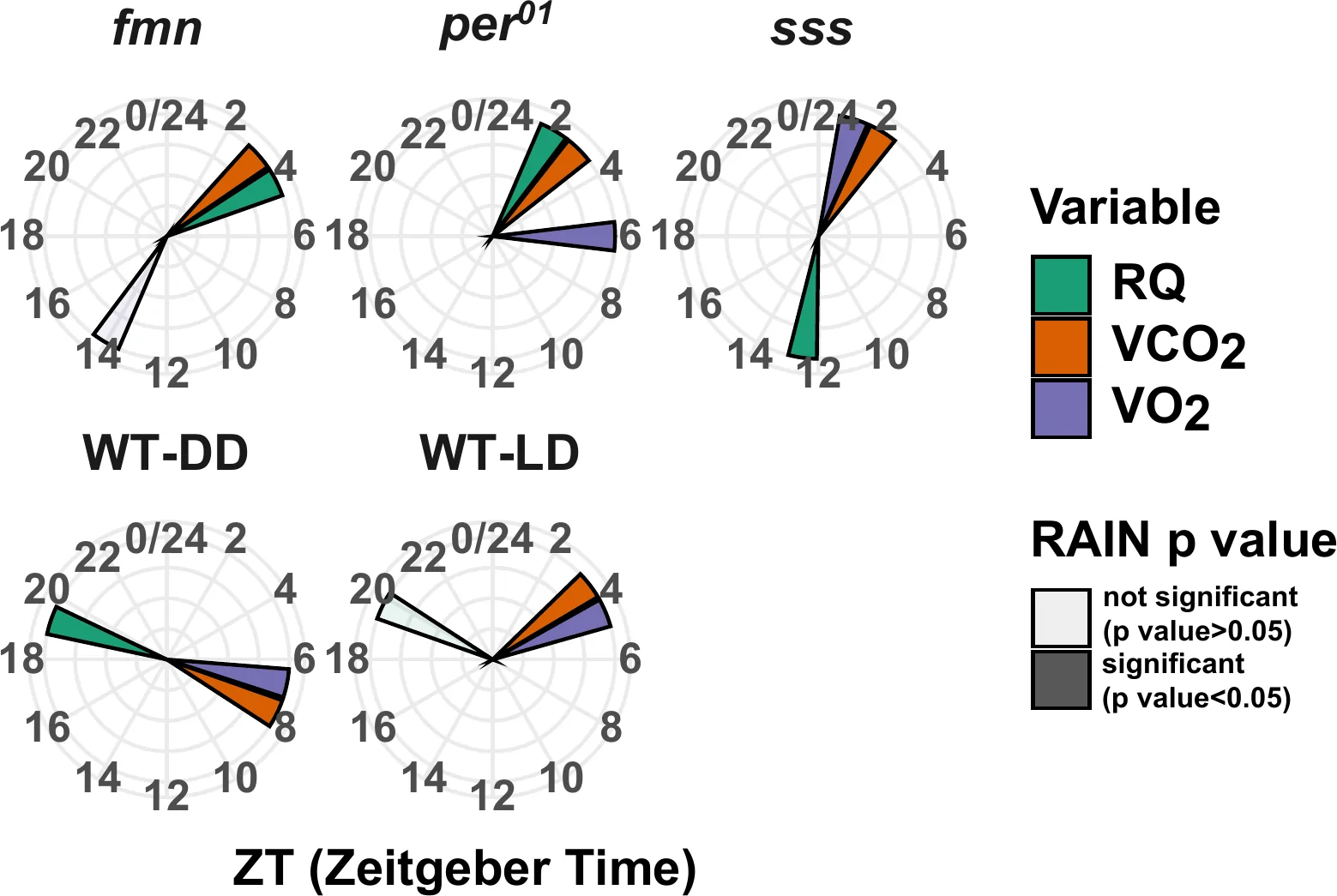
Phase distribution of respiratory rhythms across genotypes. Polar plots depicting the phase (peak timing) of rhythmic expression for carbon dioxide production (VCO_2_), oxygen consumption (VO_2_), and respiratory quotient (RQ) over a 24 hr cycle (Zeitgeber time [ZT]0–24) for each genotype. Genotypes include wild-type in light-dark (WT-LD) and constant darkness (WT-DD), short-sleep mutants *fumin* (*fmn*) and *sleepless* (*sss*), and circadian mutant *per^01^.* Statistical significance of rhythmicity was determined using the RAIN algorithm: darker-colored bars indicate significant rhythms (p<0.05), and lighter-colored bars indicate non-significant rhythms (p>0.05). Where applicable, plotted values are presented as mean ± SEM. Source data are the same continuous respirometry recordings shown in Figure 1; Figure 3 presents phase/peak-timing analysis derived from the rhythmicity analysis.

Furthermore, to account for potential differences in body size that could confound VCO_2_ and VO_2_ measurements, fly weights were recorded for each genotype before respirometry. Notably, *fmn* and *sss* mutants weighed significantly less than WT controls, whereas *per^01^* flies had comparable weights (Figure 1g). Accordingly, VCO_2_ and VO_2_ values were normalized to fly weight, and the genotype-specific differences remained robust after normalization (Figure 1h–i). These data confirm that the observed metabolic differences are not attributable to body size but reflect inherent alterations in metabolic fuel utilization. Figures 1—3, Figure 1—figure supplement 4 are derived from the same underlying continuous respirometry recordings but present different analytical summaries: Figure 1 shows 5-min-binned time-course traces and 24 hr averages, Figures 2 and 3 present rhythmicity and phase analyses, and Figure 1—figure supplement 4 presents day (ZT0–12) versus night (ZT12–24) averages.

### Diurnal variation in CO_2_ production, O_2_ consumption, and RQ across genotypes

To investigate how sleep and diurnal regulation influence temporal patterns of metabolism, we measured carbon dioxide production (VCO_2_), oxygen consumption (VO_2_), and RQ across day and night phases in wild-type flies maintained under light-dark cycles (WT-LD), wild-type flies kept in constant darkness (WT-DD) to assess free-running circadian regulation in the absence of external light-dark cues, short-sleep mutants (*fumin* [*fmn*] and *sleepless* [*sss*]), and the circadian clock mutant *period*^01^ (*per*^01^). The average values of VCO_2_, VO_2_, and RQ during the day (ZT0–12) and night (ZT12–24) are presented in Figure 1—figure supplement 4.

The *sss* mutant exhibited the highest metabolic rates among all genotypes, with significantly elevated VCO_2_ and VO_2_ during both day and night relative to WT-LD (p<0.001). A pronounced day-night difference in *sss* further indicated a strong phase-dependent increase in respiratory activity (Figure 1—figure supplement 4). The *fmn* mutant also showed significantly elevated VCO_2_ and VO_2_ compared to WT-LD (p<0.001), with a detectable but less marked diurnal variation than in *sss* (Figure 1—figure supplement 4). WT-LD flies exhibited a robust diurnal pattern, with significantly higher respiratory rates during the day (p<0.001), consistent with LD-associated temporal regulation of energy expenditure (Figure 1—figure supplement 4).

The circadian mutant *per^01^*, lacking a functional molecular clock, displayed an attenuated and phase-altered respiratory pattern. Both VCO_2_ and VO_2_ were significantly reduced during the day (p<0.05), with no significant changes at night, indicative of disrupted or misaligned metabolic patterning (Figure 1—figure supplement 4). WT-DD flies, maintained in constant darkness, exhibited the lowest overall respiratory activity. Despite the absence of environmental light cues, VCO_2_ and VO_2_ retained significant day-night differences (Figure 1—figure supplement 4), consistent with persistent free-running circadian control in constant darkness (with altered amplitude and/or phase relative to LD).

### Diurnal and circadian variations in CO_2_ production, O_2_ consumption, and RQ across genotypes

To evaluate genotype-dependent 24 hr rhythmic regulation of metabolism, we assessed rhythmicity in carbon dioxide production (VCO_2_), oxygen consumption (VO_2_), and RQ in wild-type flies under light-dark conditions (WT-LD), wild-type flies in constant darkness (WT-DD), short-sleep mutants (*fumin* [*fmn*] and *sleepless* [*sss*]), and the circadian clock mutant *period^01^* (*per^01^*). All mutant strains were assayed under LD. Time-course data were analyzed using JTK CYCLE and RAIN algorithms, with rhythmicity classified as statistically significant (p≤0.05), trending (0.05<p<0.1), or not significant (p≥0.1). Rhythmic phase was estimated using JTK lag values (Figures 2 and 3, Table 1).

**Table 1.** Rhythmicity metrics for respiratory parameters across genotypes.

Genotype	RQ JTK lag	RQ JTK period	RQ RAIN p-value	VCO_2_ JTK lag	VCO_2_ JTK period	VCO_2_ RAIN p-value	VO_2_ JTK lag	VO_2_ JTK period	VO_2_ RAIN p-value
WT-LD	19.75	20	0.12	4	24	1.1143e^-05^	4	24	0.001
*fumin*	4.25	22.5	9.54e^-08^	3.25	24	0.0002	14	20	0.69
*sss*	12.5	20	1.41e^-07^	2	24	1.33e^-09^	1.25	24	1.14e^-10^
*per^01^*	2	20	0.006	3	24	1.05e^-13^	6	21.5	1.82e^-07^
WT-DD	19.25	23	0.02	7.5	24	0.0002	7	24	9.4145e^-06^

VCO_2_ rhythms were statistically significant in WT-LD, WT-DD, *fmn*, *sss*, and *per^01^* (Figure 2, Table 1). These findings demonstrate that VCO_2_ exhibits robust rhythmic oscillations under both light-dark and constant darkness conditions, and that rhythmicity persists even in the absence of a functional *period* gene. VCO_2_ peak occurred near ZT~4 in WT-LD, ZT~7.5 in WT-DD, ZT~3.25 in *fmn*, ZT~3 in *per^01^*, and ZT~2 in *sss*, indicating genotype-specific shifts in respiratory phase (Figure 3, Table 1).

VO_2_ rhythmicity was statistically significant in WT-LD, WT-DD, *sss*, and *per^01^* while not significant in *fmn* (Figure 2, Table 1). These findings indicate that rhythmic regulation of oxygen consumption is evident in constant darkness and in the absence of a functional *period* gene but is reduced in a sleep mutant. VO_2_ peaked near ZT~4 in WT-LD, ZT~7 in WT-DD, ZT~1.25 in *sss*, and ZT~6 in *per^01^*, suggesting conserved phase alignment with all phases within ±3 hr of WT-LD (Figure 3, Table 1).

RQ also showed genotype-dependent rhythmicity, with significant diurnal oscillations observed in *fmn, sss*, and *per^01^*, while no significant rhythms were observed in WT-LD or WT-DD (Figure 2, Table 1). In the rhythmic genotypes, RQ peaked at ZT~4.25 in *fmn,* ZT~2 in *per^01^*, and ZT~12.5 in *sss*, indicating distinct RQ phase alignment across the sleep mutants (spread ~8 hr apart), in contrast to the conserved phase alignment of respiratory output (VO_2_) (Figure 3, Table 1).

### Temporal profiling of RQ in WT flies under light-dark conditions

**Figure 4.**
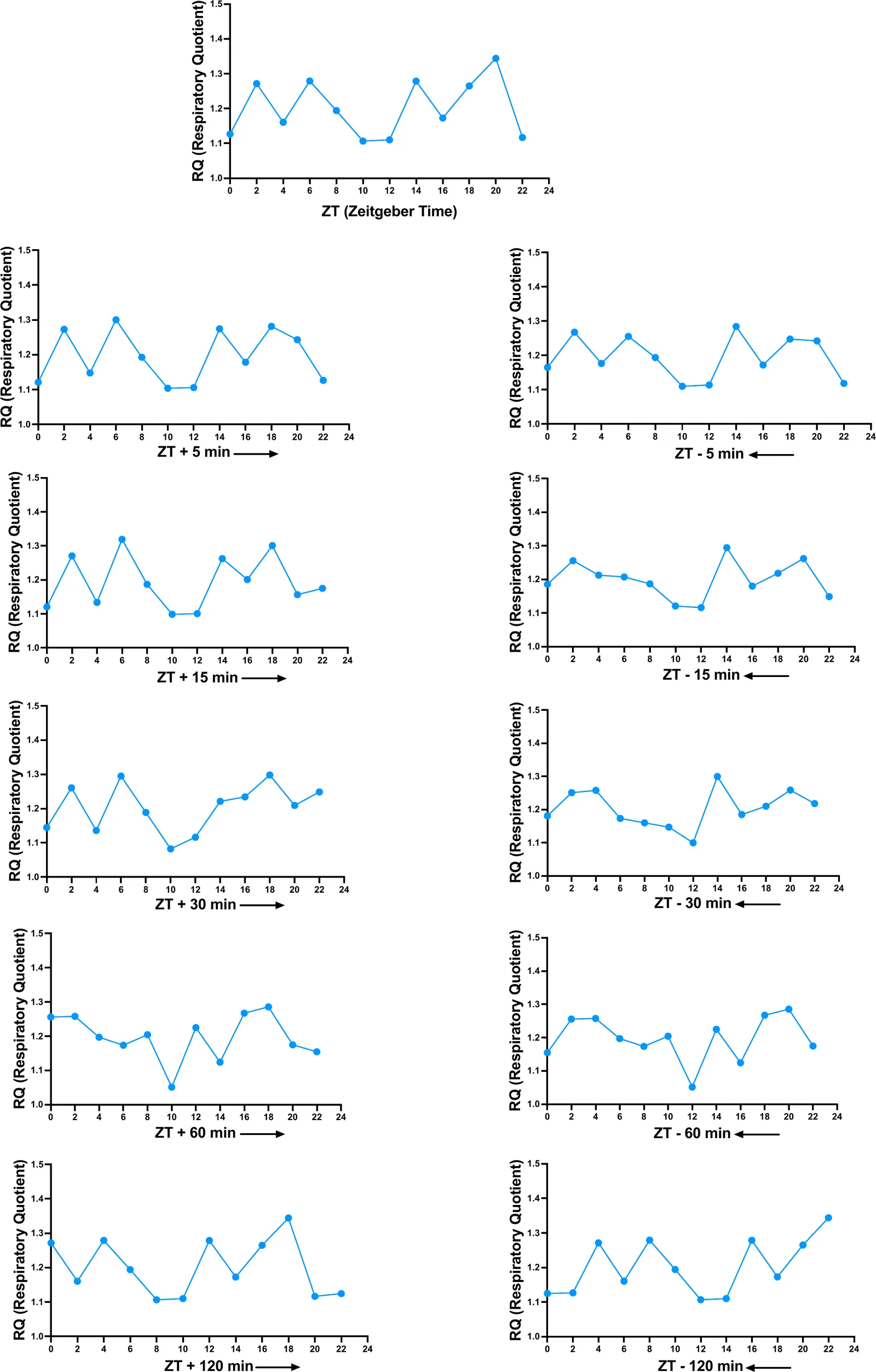
Temporal profiling of respiratory quotient (RQ) in wild-type (WT) flies under light-dark conditions. RQ was extracted every 2 hr across a 24 hr light-dark (LD) cycle in WT flies. Lag analyses were performed by advancing or delaying the RQ data by −120, –60, −30, –15, −5, +5, +15, +30, +60-, and +120 min relative to Zeitgeber time (ZT). Each panel represents the RQ profile corresponding to a specific time shift, illustrating phase-dependent respiratory dynamics across the 24 hr LD cycle. Data represent approximately 300 flies per genotype across 3 experimental days (25 flies/chamber, 4 chambers/experiment). The chamber was used as the experimental unit; *n* denotes the number of chambers. RQ rhythmicity itself was assessed from the continuous respirometry time series (Table 1); Figure 4 shows the day-night RQ pattern used as the reference for the lag-based metabolite correlations, to which the metabolomics data contribute.

RQ values corresponding to each 2 hr ZT point were extracted from the 5 min binned dataset. Building on this alignment, we explored temporal relationships by systematically shifting the RQ time series by −120, –60, −30, –15, −5, +5, +15, +30, +60, and +120 min relative to the metabolite dataset. The continuous respirometry time series showed an oscillatory day-night pattern in RQ; metabolomics was then used to relate time-matched and lagged metabolite dynamics to RQ patterns (Figure 4).

### Correlation of RQ with metabolite profiles and temporal lag analysis in WT-LD flies

**Figure 5.**
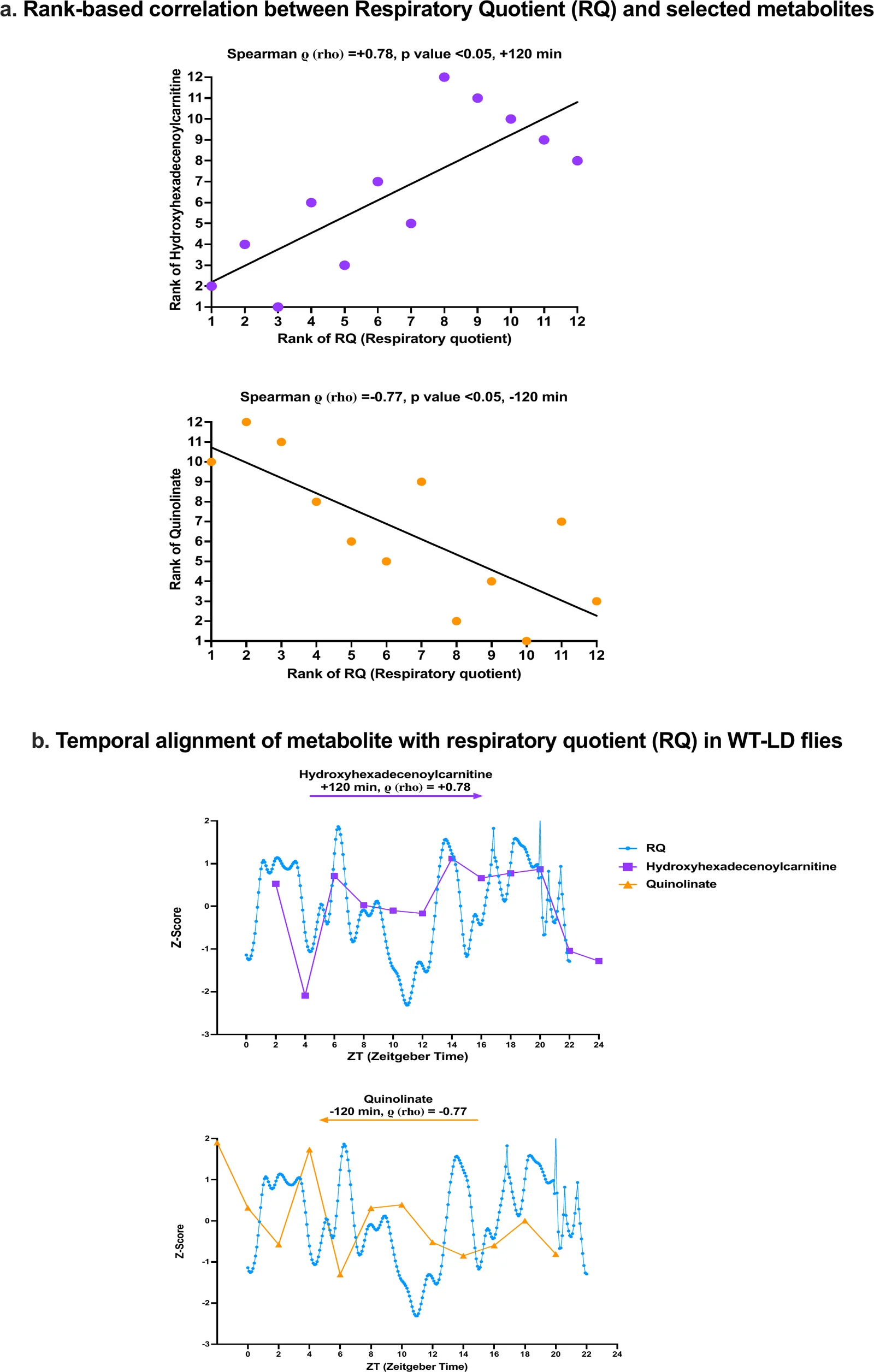
Exemplar temporal relationships between metabolites and respirometry. (**a**) Rank-based correlation between respiratory quotient (RQ) and selected metabolites. Scatter plots depict the rank-order relationships between RQ and (**A**) hydroxyhexadecenoylcarnitine and (**B**) quinolinate. Each point represents a timepoint after aligning data with respective time shifts. Spearman rank correlation coefficient (*ρ*) and corresponding p-values are indicated on each panel. A positive lag (+120 min for hydroxyhexadecenoylcarnitine) or negative lag (−120 min for quinolinate) denotes the temporal shift in RQ relative to the metabolite dataset. Statistical significance was defined as p<0.05. (**b**) Temporal alignment of metabolite with RQ in wild-type flies under light-dark cycles (WT-LD) flies. Z-scored time series of RQ, hydroxyhexadecenoylcarnitine, and quinolinate across a 24 hr light-dark cycle. Top panel: hydroxyhexadecenoylcarnitine shows a positive lag of +120 min and strong positive correlation with RQ (*ρ*=+0.78), suggesting its rise after changes in RQ. Bottom panel: quinolinate shows a negative lag of –120 min and strong negative correlation with RQ (*ρ*=−0.77), indicating it precedes changes in RQ.

To investigate the association between RQ and metabolite in WT-LD flies, we initially performed Spearman correlation analysis (*ρ*) across a range of temporal lags (−120 to +120 min). Several metabolites demonstrated strong correlations with RQ (|*ρ*|>0.7, p<0.05), suggesting tight coupling between metabolic fluctuations and respiratory output. The full set of metabolites meeting this threshold across WT-LD, *fmn*, *sss*, *per^01^*, and WT-DD conditions, along with best lag direction, lag time, and Spearman *ρ* value and Benjamini-Hochberg (BH) false discovery rate (FDR), is provided in Supplementary file 1A. To minimize the influence of outliers and enhance data visualization, selected correlation patterns were subsequently presented as rank-based scatter plots. Representative examples are shown in Figure 5a to illustrate the metabolite-RQ correlation and lag-analysis workflow. Hydroxyhexadecenoylcarnitine and quinolinate are among the strongest positively and negatively lagged RQ-correlated metabolites in WT-LD (*ρ* = +0.78 at +120 min lag and *ρ* = −0.77 at −120 min lag; Supplementary file 1A), illustrating the two opposite lag directions of the workflow. We define ‘anticipatory’ as metabolite changes that precede the associated respiratory shift (negative lag) and ‘reactive’ as changes that follow or coincide with the respiratory shift (positive lag), and we use these terms consistently throughout. To further characterize these associations, metabolites were categorized based on the timing of their peak changes relative to RQ fluctuations, exhibiting either positive or negative lag. To enable direct comparison of temporal dynamics between RQ and metabolite profiles across ZT0–24, z-scoring was performed to normalize differences in absolute magnitude and measurement units. Within this framework, a positive lag indicates that metabolite changes occur after shifts in RQ, requiring the metabolite profile to be shifted forward (rightward) along the time axis for optimal alignment. Conversely, a negative lag signifies that metabolite changes precede RQ fluctuations, necessitating a backward (leftward) shift of the metabolite profile to achieve alignment (Figure 5b).

### Metabolite response dynamics relative to RQ and functional pathway enrichment of RQ-associated metabolites in WT-LD flies

**Figure 6.**
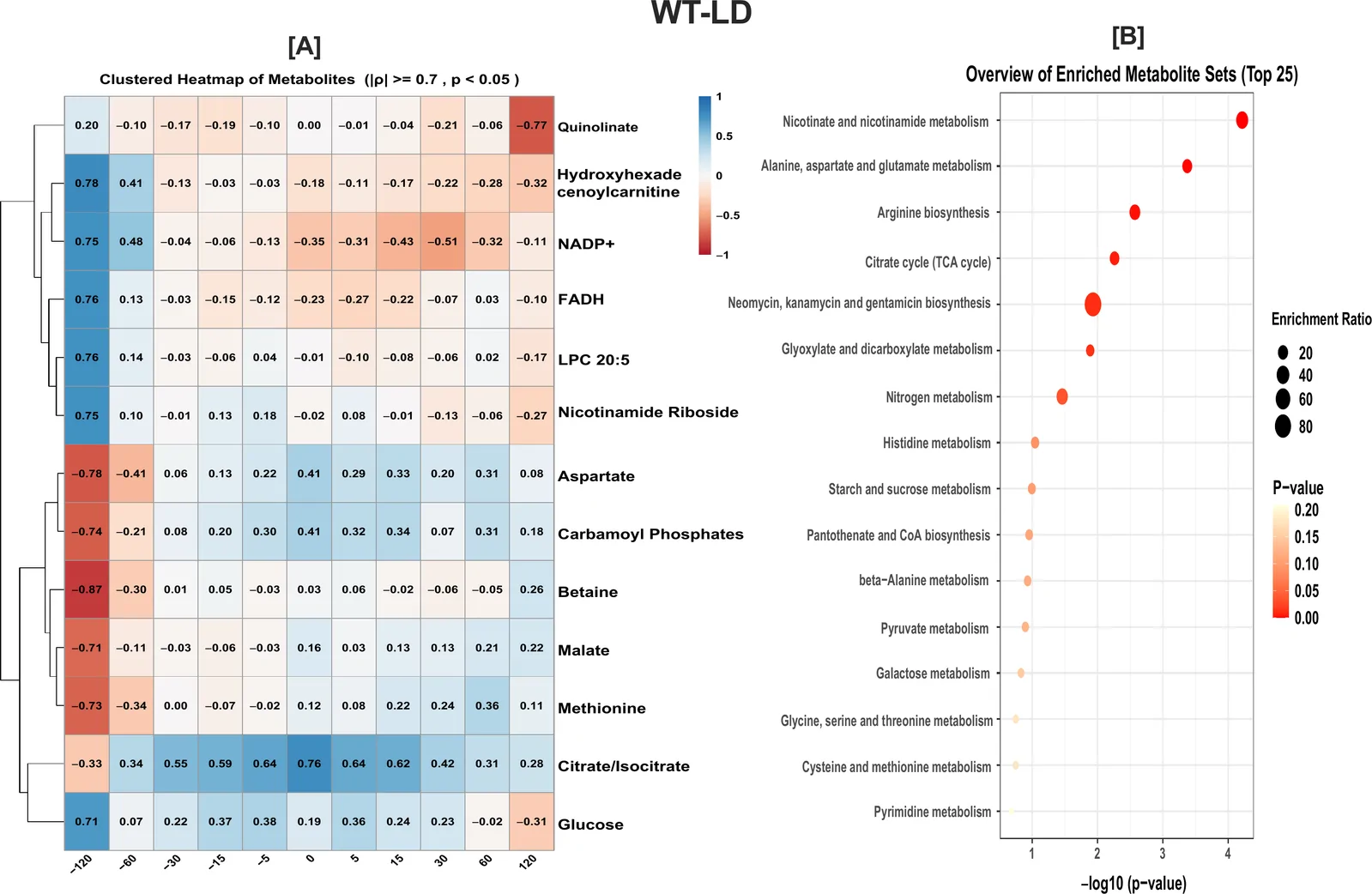
Metabolite-respiratory quotient (RQ) correlations and pathway enrichment in wild-type flies under light-dark cycles (WT-LD). (**A**) Heatmap depicting Spearman correlations (|*ρ*|>0.7, p<0.05) between RQ and metabolites across the 24 hr LD cycle in wild-type flies maintained under light-dark (LD) conditions. Metabolites were clustered based on correlation patterns, highlighting groups with similar temporal associations with respiratory activity. (**B**) Pathway enrichment analysis of significantly correlated metabolites, illustrating metabolic pathways most closely linked to respiratory dynamics. Pathways with a p-value less than 0.05 were considered statistically significant.

To further explore the temporal relationship between the metabolites and RQ, we generated a clustered heatmap of metabolites significantly correlated with RQ. Metabolites were filtered based on statistical significance (|*ρ*|>0.7, p<0.05 at any timepoint), and their temporal profiles were realigned relative to respiration, anchoring RQ at *t*=0 (Figure 6, Supplementary file 1B). This flipping step was essential to standardize the visualization of metabolites either preceding or following respiratory changes, facilitating clearer interpretation of biological timing relationships.

To identify key metabolic pathways associated with diurnal regulation of respiration, we next performed pathway enrichment analysis on the RQ-correlated metabolites using MetaboAnalyst. Enriched pathways were identified at a significance threshold of p<0.05. WT-LD-specific pathway enrichment results are presented in Figure 6. Consistent with these findings, WT flies under LD conditions exhibited diurnal-regulated metabolic rhythms, characterized by coordinated activity in amino acid metabolism, redox balance, and energy-generating pathways such as the TCA cycle and glyoxylate metabolism.

### RQ-linked metabolic shifts and pathway enrichment in sleep mutants

**Figure 7.**
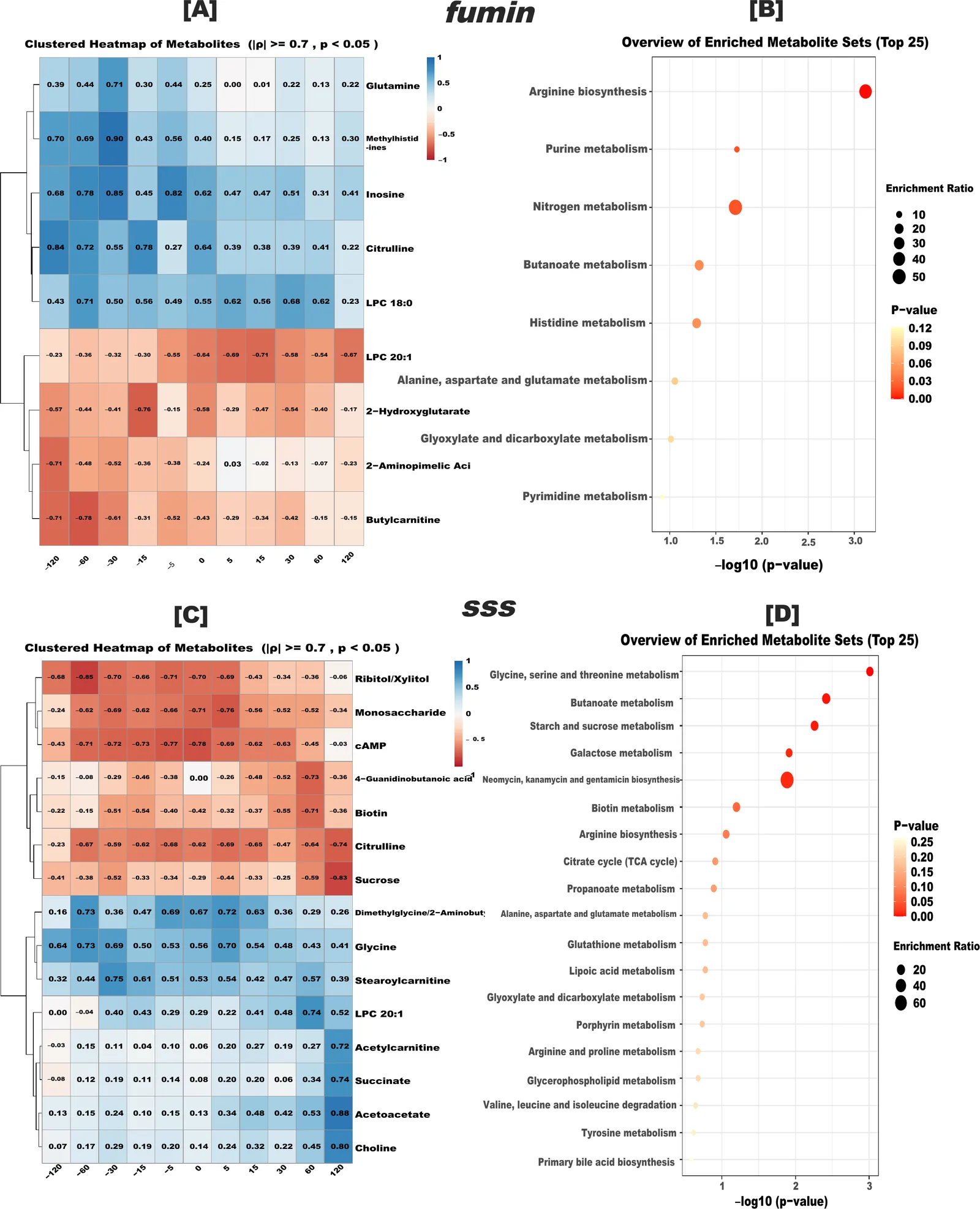
Correlation of metabolites with respiratory quotient (RQ) and pathway enrichment in short-sleep mutants. (**A, C**) Heatmaps showing Spearman correlations (|*ρ*|>0.7, p<0.05) between RQ and metabolites in the short-sleep mutants *fmn* (**A**) and *sss* (**C**). (**B, D**) Pathway enrichment analyses of metabolites significantly correlated with RQ in *fmn* (**B**) and *sss* (**D**). Pathways with p-values less than 0.05 were considered statistically significant.

We applied the same analysis method to sleep mutant flies (*fmn* and *sss*) to identify metabolic pathways associated with altered respiratory rhythms. Metabolites significantly correlated with RQ (|*ρ*|>0.7, p<0.05) were identified, and their temporal profiles were realigned relative to respiration by anchoring RQ at *t*=0. Clustered heatmaps were generated to visualize metabolite response dynamics, and pathway enrichment analysis of RQ-associated metabolites was conducted using MetaboAnalyst (p<0.05) (Figure 7). This analysis revealed that sleep mutants exhibit genotype-specific metabolic disruptions, implicating mitochondrial and energy-related pathways. In *fmn* flies, alterations were observed in arginine biosynthesis, purine and nitrogen metabolism, and butanoate pathways, suggestive of dysregulated nitrogen balance and mitochondrial dysfunction. In contrast, *sss* mutants showed perturbations in glycine, serine, and threonine metabolism, as well as carbohydrate and antibiotic biosynthetic pathways, reflecting altered carbon utilization and mitochondrial-associated metabolic stress.

### RQ-linked metabolic shifts in circadian mutants and WT flies in constant darkness

**Figure 8.**
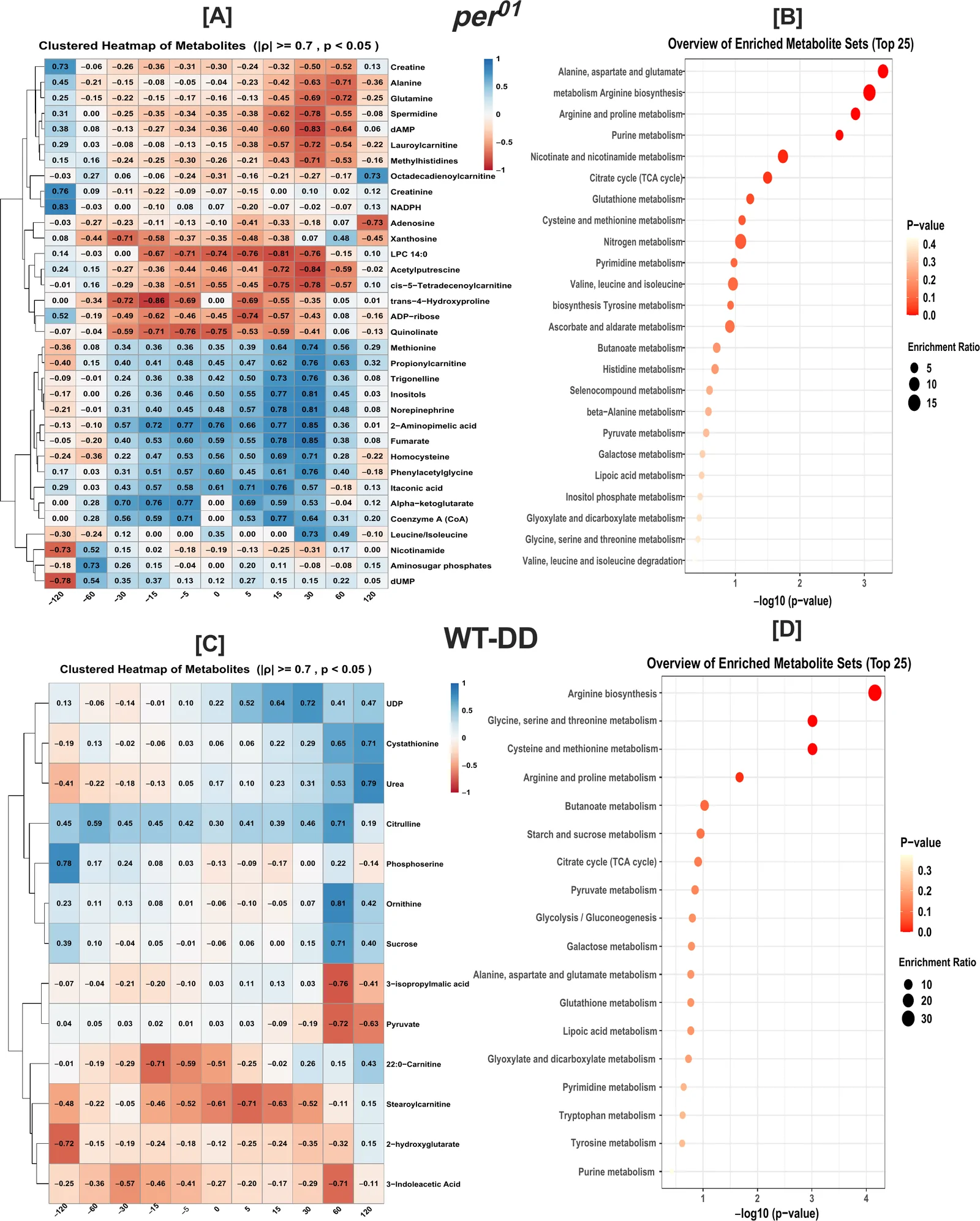
Correlation of metabolites with respiratory quotient (RQ) and pathway enrichment in circadian mutant and wild-type flies in constant darkness. (**A, C**) Heatmaps displaying Spearman correlations (|*ρ*|>0.7, p<0.05) between RQ and metabolites in the circadian mutant *per^01^* (**A**) and wild-type flies maintained in constant darkness (WT-DD) (**C**). (**B, D**) Pathway enrichment analyses of metabolites significantly correlated with RQ in *per^01^* (**B**) and WT-DD (**D**). Pathways with p-values less than 0.05 were considered statistically significant.

Circadian clock mutant flies (*per^01^*) and wild-type flies maintained under constant darkness (WT-DD) were analyzed using the same approach. We used constant darkness (DD) to assess free-running circadian regulation in the absence of external light-dark cues. Metabolites exhibiting strong correlations with RQ (|*ρ*|>0.7, p<0.05) were identified and temporally aligned by anchoring RQ at *t*=0. Clustered heatmaps were constructed to visualize the dynamic responses of these metabolites, and pathway enrichment analysis of RQ-associated metabolites was performed using MetaboAnalyst (p<0.05) (Figure 8). Both *per^01^* and WT-DD flies exhibited disrupted temporal coordination of mitochondrial metabolism. *per^01^* mutants showed dysregulation in amino acid metabolism, purine turnover, and redox-associated pathways such as glutathione and nicotinate metabolism, consistent with impaired redox buffering and energy imbalance. In contrast, WT-DD flies exhibited alterations in sulfur- and nitrogen-containing amino acid pathways, indicative of desynchronized but intact metabolic cycling in the absence of external light cues.

### Gut tissue respirometry reveals elevated baseline mitochondrial respiration in *fmn* and *per*^*01*^ mutants

**Figure 9.**
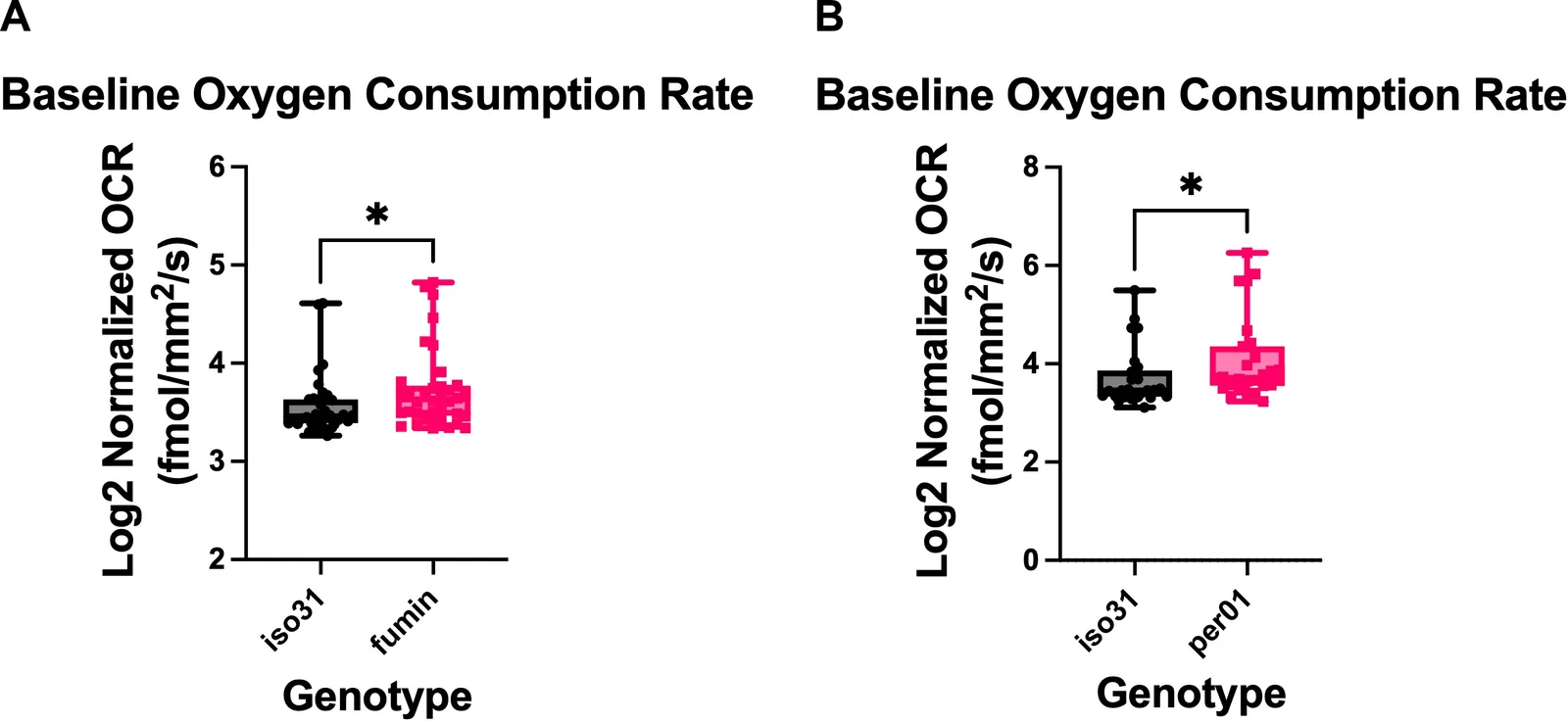
Baseline oxygen consumption in isolated gut tissue from *fmn* and *per*^*01*^ mutants. Baseline oxygen consumption rate (OCR, fmol/mm^2^/s) measured by tissue respirometry in individual dissected guts from the short-sleep mutant *fmn* (**A**) and the circadian-clock mutant *per^01^* (**B**) relative to *iso^31^* genetic-background controls. For each gut, OCR was averaged over a 24 hr window beginning ~12 hr after loading, once tissue respiration had stabilized (12–36 hr). To combine independent runs, OCR was normalized to the median of the *iso^31^* controls within each run and log2(x+10)-transformed. One *iso^31^* gut in the third run of the *per^01^* experiment was excluded as an outlier; no other values were removed. Baseline OCR differed significantly between *iso^31^* and each mutant (Mann-Whitney test, p<0.05; pooled across 3 independent runs; *fmn* vs *iso^31^*, *n*=12 vs 12; *per^01^* vs *iso^31^*, *n*=12 vs 11 after the *iso^31^* outlier exclusion).

Because pathway-level metabolomics implicated mitochondrial pathways in sleep and circadian mutants, we directly assayed mitochondrial respiration by measuring baseline oxygen consumption rate (OCR) in dissected gut tissue from *fmn* and *per^01^* flies relative to iso controls. Both *fmn* and *per^01^* guts showed significantly elevated baseline OCR (*fmn* vs *iso^31^*, *n*=12 vs 12; *per^01^* vs *iso^31^*, *n*=12 vs 11 across 3 runs; Mann-Whitney test, p<0.05; Figure 9A and B). Because only baseline OCR was measured, we interpret this as altered (elevated) baseline mitochondrial respiration rather than reduced capacity or a specific coupling defect (Figure 9).

## Discussion

### Temporal misalignment alters fuel utilization and respiratory rhythms

Our integrative approach, combining whole-organism respirometry (Brown et al., 2022; Stahl et al., 2017) with LC-MS-based metabolomics (Malik et al., 2024; Tennessen et al., 2014; Cirelli, 2009; Johnson et al., 2016; Patti et al., 2012; Wishart, 2016), reveals how sleep and circadian disruptions distinctly alter energy metabolism in *D. melanogaster*. By comparing WT flies with mutants affecting sleep (*fumin* and *sleepless*) (Kume et al., 2005; Koh et al., 2008; Wu et al., 2008; Shi et al., 2014) and circadian clock function (*per^01^*) (Wu et al., 2010), we have captured genotype-specific differences in respiratory dynamics and substrate usage across daily cycles under tightly controlled environmental conditions. These findings establish a functional framework for understanding how behavioral state and clock integrity shape metabolic homeostasis.

Across the manuscript, the central takeaway is that WT flies under LD exhibit anticipatory alignment of fuel selection with time of day, whereas short-sleep mutants (*fmn, sss*) and clock-disrupted flies (*per^01^*) show reactive or misaligned metabolism under our conditions. We therefore focus the Discussion on loss of temporal coordination between respiratory output and pathway-level metabolism, rather than reiterating rate changes alone.

Metabolites in WT-LD typically showed peak correlations preceding respiratory changes (negative lag), reflecting anticipatory diurnal metabolic regulation under LD conditions. In contrast, mutants displayed a shift to reactive correlations (positive lag), indicating loss of temporal synchronization and proactive metabolic adjustments. These phenotypes parallel mammalian systems, where sleep loss and circadian misalignment are linked to elevated basal metabolic rate, a shift toward carbohydrate oxidation and lipid/protein catabolism, and blunted, phase-shifted respiratory oscillations (Sharma and Kavuru, 2010; Ho et al., 2016; Jung et al., 2011). In our flies, the short-sleep mutants show the analogous catabolic shift (*fmn* RQ 1.09; *sss* RQ 0.94), and *per^01^* and WT-DD show dampened, phase-shifted respiratory rhythms (Woller and Gonze, 2021; Dibner and Schibler, 2015). These conserved responses underscore the translational value of *Drosophila* for dissecting the interplay between sleep, circadian timing, and metabolic regulation, while physiological differences between flies and mammals caution against direct mechanistic extrapolation.

### WT flies exhibit diurnal regulation of biosynthesis and redox balance under LD cycles

In WT flies maintained under a 12:12 LD cycle, RQ-correlated metabolites revealed tightly coordinated diurnal regulation of biosynthetic and redox pathways. An increase in nicotinate and nicotinamide metabolism – highlighted by changes in quinolinate, nicotinamide riboside, and L-aspartic acid – suggests a direct link between mitochondrial respiration and NAD^+^ biosynthesis (O’Neill and Reddy, 2011; Nakahata et al., 2009). These NAD^+^ precursor changes occur before respiration increases (negative lag), highlighting anticipatory NAD^+^ regulation. Concurrent alterations in alanine, aspartate, and glutamate metabolism (via citric acid and carbamoyl phosphate) and arginine biosynthesis indicate dynamic alignment of amino acid turnover with energy demand and nitrogen disposal (Green et al., 2008; Rey et al., 2018). RQ-associated enrichment of citrate cycle intermediates such as malate and citric acid further supports diurnal gating of oxidative phosphorylation (Peek et al., 2013). Elevated levels of D-glucose were mapped to the KEGG pathway for neomycin, kanamycin, and gentamicin biosynthesis. While *Drosophila* does not synthesize these antibiotics, the enrichment likely reflects the presence of shared carbohydrate intermediates (e.g. glucose, glucose-6-phosphate, UDP-glucose) common to multiple biosynthetic processes. This mapping is more indicative of altered carbohydrate and amino sugar metabolism, or potentially reflects microbial contributions, rather than actual antibiotic production by the host (Sharon et al., 2010; Wong et al., 2014). Additional contributions from glyoxylate and dicarboxylate metabolism reinforce the role of anaplerotic flux and redox cycling (Stenvers et al., 2019). Notably, persistent correlations between RQ and pantothenate precursors suggest synchronized CoA biosynthesis, potentially coordinating fatty acid oxidation and TCA cycle input (Leone et al., 2015). Together, these results demonstrate that under LD conditions, WT flies sustain temporal coordination between respiration and mitochondrial metabolism, integrating redox homeostasis, nitrogen metabolism, and substrate availability in an LD-associated diurnal manner.

### Altered amino acid metabolism and mitochondrial dysregulation in *fmn* mutants

The *fumin* (*fmn*) mutant, characterized by chronic sleep loss, exhibited genotype-specific disruptions in metabolic coordination, as revealed by metabolites whose temporal dynamics were strongly correlated with RQ. In *fmn* flies, significant RQ correlations (p<0.05) were identified in pathways, including arginine biosynthesis (citrulline, glutamine), purine metabolism (inosine, glutamine), and nitrogen metabolism, suggesting a decoupling of amino acid turnover and nucleotide cycling from respiratory demand (Donlea et al., 2014; Liu et al., 2016). Glutamine’s strong association with RQ highlights its central role in buffering nitrogen flux and supporting biosynthetic processes under energetic stress (Brosnan, 2000). Correlated dynamics of 2-hydroxyglutarate, a key intermediate in butanoate metabolism, point to impaired TCA cycle flux and redox imbalance (Reinecke et al., 2009). Likewise, RQ-associated shifts in methylhistidine suggest altered histidine metabolism, potentially impacting mitochondrial protein turnover and methylation processes. Acylcarnitine, indicators of lipid oxidation, showed consistently strong RQ correlations across genotypes, most pronounced in *fmn*, underscoring lipid metabolism’s role in respiratory coupling. Particularly in mutants, increased reliance on fatty acid oxidation may reflect compensatory responses to disrupted carbohydrate metabolism and sustained energetic demands. Together, these findings indicate a failure in aligning substrate oxidation with mitochondrial respiratory output in *fmn* mutants. Building on prior evidence of mitochondrial stress in sleep-deprived *fmn* flies (Vaccaro et al., 2020), our integrative respirometry-metabolomics analysis reveals disrupted coupling between energy metabolism and amino acid pathways, underscoring the physiological cost of chronic sleep loss on mitochondrial homeostasis. These interpretations are further supported by gut tissue respirometry showing elevated baseline mitochondrial respiration (OCR) in *fmn* relative to iso31 controls. Together with prior evidence of ROS accumulation (oxidative stress) in *fmn* (Vaccaro et al., 2020), these functional data indicate that chronic sleep loss in *fmn* is associated with altered mitochondrial respiration.

### Disrupted carbon and amino acid metabolism in *sss* mutants

The *sleepless* (*sss*) mutant displayed significant disruptions in carbon and amino acid metabolism, as indicated by strong correlations between RQ and metabolites involved in glycine, serine, and threonine metabolism (choline, dimethylglycine, glycine), butanoate metabolism (acetoacetate, succinate), and carbohydrate pathways (Koh et al., 2008). Notably, RQ-associated shifts in choline and dimethylglycine suggest dysregulation of one-carbon metabolism and methyl group transfer, which can impair mitochondrial function and epigenetic stability (Mentch et al., 2015; Locasale, 2013). Correlated dynamics of acetoacetate, a ketone body often found in starvation, and succinate (both key intermediates of butanoate metabolism) are consistent with altered TCA cycle input and redox balance (Owen et al., 2002; Finkel et al., 2015). In parallel, strong RQ correlations with sucrose and D-glucose (from both starch/sucrose and galactose metabolism) indicate disrupted carbohydrate processing and inefficient substrate utilization (Keene et al., 2010). cAMP and biotin were also found to associate with respiration, both unique in metabolic regulation through altered neuromodulatory signaling and cofactor metabolism. These findings suggest that *sss* mutants exhibit altered metabolic routing in response to respiratory demand, shifting substrate utilization toward carbohydrate and amino acid pathways under sleep-deprived conditions, consistent with metabolic stress responses observed in other models of sleep loss (Hatori et al., 2012).

### Loss of temporal coupling of mitochondrial metabolism in clock mutants

In *period* (*per^01^*) mutants lacking a functional circadian clock, the temporal coordination between metabolism and respiration was disrupted across nearly every measured metabolite through intermittent strong correlations with respiration, reflecting broad metabolic chaos (Bass and Takahashi, 2010; Hardin, 2005). Strong correlations with RQ were observed in alanine, aspartate, and glutamate metabolism (L-alanine, glutamine, fumarate, oxoglutarate) and arginine biosynthesis, suggesting inefficient routing of amino acid-derived substrates into mitochondrial energy production (Rey et al., 2018). Delayed RQ-associated dynamics of glutamine, oxoglutarate, and fumarate indicate a mismatch between substrate availability and respiratory output (Panda, 2016). Additionally, metabolites from arginine and proline metabolism (creatine, spermidine, *N*-acetylputrescine, 4-hydroxyproline) exhibited altered RQ correlations, suggesting impaired mitochondrial redox buffering and compromised integrity (Wilking et al., 2013). Perturbations in purine metabolism – including adenosine, deoxyadenosine monophosphate, xanthosine, and ADP-ribose – further point to dysregulated ATP turnover and nucleotide imbalance. Correlations with quinolinic acid and niacinamide reflect altered nicotinate and nicotinamide metabolism, implicating disrupted NAD^+^ biosynthesis and redox state (Nakahata et al., 2009). Additional RQ-associated changes in NADPH and TCA intermediates (fumarate, oxoglutarate) align with impaired glutathione metabolism and altered mitochondrial metabolism. Gut tissue respirometry in *per^01^* showed elevated baseline OCR. Because only baseline respiration was measured, we interpret this as altered (elevated) baseline mitochondrial respiration – which can reflect inefficient or uncoupled respiration, or compensatory upregulation, rather than increased functional capacity – consistent with the metabolomic signatures of disrupted redox balance and mitochondrial metabolism. Microbial-derived metabolites (trigonelline, phenylacetylglycine) were also identified in the correlation analysis, suggesting potential gut microbiome interactions that become evident in circadian disruption. Together, these findings suggest that the loss of circadian timing in *per^01^* mutants leads to widespread uncoupling of substrate utilization and mitochondrial respiration. This breakdown in temporal regulation disrupts energy homeostasis, highlighting the essential role of the circadian clock in coordinating metabolic flux with respiratory demand (Bass and Takahashi, 2010; Peek et al., 2013; Kriebs et al., 2017).

### Metabolic desynchrony and redox imbalance in WT flies under constant darkness

In wild-type flies maintained under constant darkness (DD), the absence of environmental light cues led to disrupted temporal alignment between mitochondrial respiration and metabolic pathways (Allada and Chung, 2010; Dubowy and Sehgal, 2017). Generally, we note a shift toward reactive metabolic adjustments (positive lags) compared to WT-LD instead of anticipatory preparation, indicating reduced metabolic efficiency. RQ-correlated metabolites showed significant enrichment in arginine biosynthesis (citrulline, ornithine, urea), glycine, serine, and threonine metabolism (L-cystathionine, phosphoserine, pyruvic acid), and cysteine and methionine metabolism-indicating altered integration of nitrogen and sulfur amino acid metabolism with respiratory activity (Green et al., 2008). Notably, the convergence of pyruvic acid across multiple pathways suggests dysregulated entry points into the TCA cycle, while the consistent association of L-cystathionine highlights impaired redox buffering and methylation capacity (Dallmann et al., 2014; Mentch and Locasale, 2016). Altered correlations in arginine and proline metabolism (ornithine, pyruvic acid) further underscore compromised coupling between amino acid turnover and mitochondrial energy production (Rhoades et al., 2018). Altered lipid metabolism and increased nitrogen catabolism are likely compensatory mechanisms in the absence of environmental cues. These findings build on previous reports of circadian desynchrony by providing functional evidence that environmental light cues contribute to coherent coordination between respiration and key metabolic circuits (Rey et al., 2018; Hughes et al., 2009). The resulting metabolic misalignment under constant darkness emphasizes the circadian clock’s dependence on external entrainment to regulate energy homeostasis at the organismal level (Stenvers et al., 2019). Our DD data show that endogenous free-running circadian regulation persists but that removing external light-dark cues degrades the temporal coordination between respiration and metabolism, indicating that external entrainment normally strengthens this coordination under our conditions; however, we acknowledge that the coupling may be different in mammals. We also restrict our conclusions to the genotypes and conditions tested (*fmn, sss*, and *per^01^*) and do not generalize these effects to acute sleep deprivation, which was not performed in this study. Accordingly, the ‘metabolic misalignment’ observed in constant darkness (DD) likely results from a decrease in synchrony due to the removal of external light:dark cues. We also acknowledge the limits of this replication: with *n*=12 chambers per genotype, statistical power to detect small-magnitude differences and subtle phase shifts is limited, and negative calls (e.g. arrhythmicity) should be interpreted with this caveat.

Locomotor activity and feeding could not be measured during the respirometry recordings, so genotype differences in respiratory parameters should be interpreted with caution. To assess whether behavioral timing could account for these differences, we compared our respiratory rhythms with the feeding and activity rhythms reported for these genotypes in our previous work (Malik et al., 2026): WT feeding peaked at ZT~3.25 and *fmn* feeding was phase-delayed to ZT~4.5, while *fmn* also showed elevated locomotor activity, particularly during the dark period. In our data the *fmn* VCO_2_ rhythm peaks at ZT~3.25 (RQ at ZT~4.25), so the respiratory peak slightly precedes the feeding peak; the phase of the *fmn* respiratory rhythm is therefore not driven by feeding, although the elevated activity of *fmn* may contribute to its higher overall metabolic rate. Feeding and activity were not measured for *sss*.

Because only males were analyzed, our conclusions may not generalize to females, which can show sex-specific metabolic physiology. Inclusion of female flies and direct sex comparisons across LD and DD conditions will be an important future direction. More specifically, females carry a higher reproductive and biosynthetic load (egg production) that typically raises metabolic rate and can shift RQ toward lipogenesis and alter the amplitude and phase of diurnal respiratory rhythms; females might therefore show larger or differently timed effects than the males studied here.

### Conclusions

This study demonstrates that sleep loss and circadian disruption impair metabolic homeostasis in *D. melanogaster* via distinct yet converging mechanisms. Wild-type flies under light-dark cycles (WT-LD) maintained coordinated anticipatory respiratory and metabolic rhythms, while sleep mutants (*fmn*, *sss*) and circadian-disrupted flies (*per^01^*) showed altered substrate utilization, redox imbalance, and uncoupling of mitochondrial pathways, likely reactive to respiration. These findings highlight the essential role of both sleep and circadian timing in regulating energy metabolism. The presence of neuromodulatory metabolites (e.g. cAMP in *sleepless* mutants) and microbial-derived compounds (trigonelline, phenylacetylglycine in *per^01^* mutants) points toward additional layers of metabolic regulation influenced by neuronal activity and gut microbiota interactions, revealing more complex metabolic dysregulation in sleep-deprived and circadian-disrupted states. By combining high-resolution respirometry with targeted metabolomics, we establish *Drosophila* as a powerful model for investigating the molecular basis of sleep- and clock-related metabolic dysfunction and potential therapeutic interventions.

## Materials and methods

**Key resources table:** 

Reagent type (species) or resource	Designation	Source or reference	Identifiers	Additional information
Strain, strain background (*Drosophila melanogaster*)	Isogenic control (iso/iso31)	Laboratory background strain	iso/iso31	Wild-type isogenic control; used as WT-LD/WT-DD and as genetic-background control for gut OCR experiments.
Genetic reagent (*D. melanogaster*)	Fumin (fmn)	Kume et al., 2005	Dat[fmn] (fumin)	Dopamine transporter mutant; short-sleep/hyperactive phenotype.
Genetic reagent (*D. melanogaster*)	Sleepless (sss)	Koh et al., 2008; Chen et al., 2015; Wu et al., 2010	sss	Short-sleep mutant lacking functional Sleepless protein.
Genetic reagent (*D. melanogaster*)	Period null (per01)	Konopka and Benzer, 1971	per[01]	Circadian clock mutant lacking a functional molecular clock.
Other	MAVEn flow-through respirometry system	Sable Systems International	MAVEn by Sable Systems International (Multiple animal versatile energetics flow-through system)	Instrument. Continuous whole-fly respirometry. The MAVEn splits airflow into 16 fly-chamber channels plus one dedicated baseline channel (Materials and methods – Respirometry chambers and MAVEn system’s airflow management). Each experiment used all 16 chambers: 4 chambers×4 genotypes, 25 flies/chamber.
Other	RC respirometry chambers	Sable Systems International	RC chambers, 70 mm × 20 mm	Instrument. 70 mm × 20 mm borosilicate glass chambers; used as the 16 fly-chamber channels of the MAVEn system.
Other	Side-Trak 840 Series mass-flow controller	Sierra Instruments, Inc	Side-Trak 840 Series	Instrument. Reference-airflow control.
Other	LI-7000 CO_2_ analyzer	LI-COR Biosciences	LI-7000	Instrument. Measurement of CO_2_ production.
Other	Oxzilla II differential O_2_ analyzer	Sable Systems International	Oxzilla II	Instrument. Measurement of O_2_ consumption.
Other	Resipher System	Lucid Scientific (GA, USA)	Resipher	Instrument. Continuous OCR measurement in dissected gut tissue.
Chemical compound, drug	Drierite/desiccant	Drierite	Cat. no. 26800; CAS: 7779-18-9	Used in CO_2_ scrubbing/drying columns as described in Materials and methods.
Chemical compound, drug	Ascarite	Acros Organics	CAS: 81133-20-2	CO_2_ scrubbing material.
Other	Glass wool	Fisher Scientific	11-388	Laboratory material. Separator material in scrubbing columns.
Other	Nafion tubing	Perma Pure, LLC	TT-070	Laboratory material. Used to re-humidify scrubbed air.
Other	Bev-A-Line nonpermeable tubing	United States Plastic Corp.	56280	Laboratory material. Tubing used to connect respirometry components.
Chemical compound, drug	Magnesium perchlorate	Fisher Scientific	M54	Water-vapor scrubbing before O_2_ analysis.
Other	10 mL syringe body	Fisher Scientific	14955459	Laboratory material. Housing for small water-vapor scrubbing columns.
Other	Rubber stoppers	Fisher Scientific	14-135E	Laboratory material. Used to secure tubing to scrubbing columns.
Other	Schneider’s Media	Gibco/Life Technologies (Thermo Fisher Scientific)	Cat. no. 21720024	Cell culture medium. Used for *Drosophila* gut dissection and Resipher OCR assay (300 µL per well; Materials and methods – Gut tissue respirometry).
Chemical compound, drug	Poly-D-lysine	Gibco/Life Technologies (Thermo Fisher Scientific)	Cat. no. A3890401	96-Well plates coated with 50 µL for 30 min to promote gut tissue adhesion (Materials and methods – Gut tissue respirometry).
Software, algorithm	MAVEn Controller software	Sable Systems International		System control and respirometry data acquisition.
Software, algorithm	GraphPad Prism	GraphPad Software	Version 10; RRID:SCR_002798	Statistical analysis and outlier identification for gut OCR analysis.
Software, algorithm	Nitecap	Brooks et al., 2022	https://nitecap.org	Exploratory circadian/rhythmicity analysis framework.
Software, algorithm	RAIN	Thaben and Westermark, 2014	RAIN via Nitecap	Rhythmicity detection with FDR-adjusted p-values.
Software, algorithm	JTK_CYCLE	Hughes et al., 2010	JTK_CYCLE via Nitecap	Period and peak-phase/lag estimation.
Software, algorithm	MetaboAnalyst	MetaboAnalyst	Version 5.0; RRID:SCR_015539	Metabolic pathway enrichment and topology analysis.
Other	Human Metabolome Database (HMDB)	HMDB	HMDB; RRID:SCR_007712; https://hmdb.ca	Database. Metabolite identifiers used for pathway analysis.
Other	KEGG *Drosophila melanogaster* pathway library	KEGG	KEGG; RRID:SCR_012773; *Drosophila melanogaster* pathway library	Database. Pathway library used in MetaboAnalyst topology/enrichment analysis.
Other	Positive/negative ion-switching targeted LC-MS metabolomics platform	Yuan et al., 2012; Malik et al., 2024; Malik et al., 2018	Ion-switching LC-MS as described	Method. Previously published LC-MS workflow used for the metabolomics dataset integrated with respirometry.

### *Drosophila* strains, entrainment, and collection

*D. melanogaster* isogenic control (*iso*), two short-sleep mutants, fumin (*fmn*) (Kume et al., 2005) and sleepless (*sss*) (Koh et al., 2008; Chen et al., 2015; Wu et al., 2010), as well as a circadian clock mutant (*per^01^*) (Konopka and Benzer, 1971), were used for this study. Male flies were collected shortly after eclosion and entrained in light-dark (LD) incubators for a minimum of 3 days before diurnal (LD) time-course collection across ZT. At the time of collection, flies were between 5 and 7 days old. WT isogenic control flies were either maintained under 12 hr:12 hr light versus dark conditions (WT-LD) or placed in constant darkness (WT-DD) for at least 24 hr prior to collection to examine light-independent rhythms. Mutant strains (*fmn, sss*, and *per^01^*) were maintained under LD cycles. All flies were reared on a standard cornmeal/molasses medium at 25°C. ZT0 was designated as lights-on, with lights-off occurring at ZT12 (12/12 LD).

Only male flies were used for all respirometry experiments and for integration with the metabolomics dataset. This was done to reduce variability introduced by female reproductive status (e.g. mating/egg production) and associated metabolic differences, enabling a clearer comparison across genotypes and lighting conditions.

### Respirometry setup

All experiments were performed using groups of 25 flies per chamber. To account for genotype-specific differences in body size, each group of 25 flies (*WT, fmn, sss*, and *per^01^*) was weighed prior to respirometry, and these weights were used to normalize VCO_2_ and VO_2_ values for accurate comparison. Each chamber was provisioned with 2 mL of fly food (2% agar, 5% sucrose) to sustain the flies during the 24 hr continuous recording period. Control chambers containing only food, without flies, were included to account for background microbial metabolism. To simulate natural microbial inoculation, flies were briefly introduced into these control chambers and then removed before data acquisition began. Each genotype measurement represents an average of ~300 flies (25 flies/chamber×4 chambers/experiment×3 experimental days), with the chamber as the experimental unit. No formal prospective power calculation was performed; replicate number was based on the 16-chamber capacity of the MAVEn system and empirical optimization showing that 25 flies per chamber produced a clear, reliable respiratory signal. Experimental groups were not randomized because they were genetically defined genotypes, and investigators were not blinded to genotype during experimental setup or analysis; respirometry and gut OCR measurements were acquired instrumentally rather than by subjective scoring.

The respirometry system continuously measured CO_2_ production and O_2_ consumption using a flow-through MAVEn system. It consisted of five main components: (1) a zero-grade air source and scrubbing column to remove residual CO_2_, (2) mass-flow controllers to regulate airflow, (3) Sable Systems RC chambers to house the flies, (4) the MAVEn system to direct air sequentially from each chamber, and (5) gas analyzers for measuring CO_2_ and O_2_ concentrations. The system was calibrated every three to five trials using 100% N_2_ (CO_2_=0 ppm) and a known CO_2_ standard to ensure measurement accuracy.

#### Zero-grade air supply and scrubbing system

Zero-grade compressed air (<0.1 ppm hydrocarbons) was first passed through a custom-built scrubbing column (#26800, Drierite) to remove residual CO_2_. The column was packed with an inner layer of Ascarite (#81133-20-2, Acros Organics) flanked by two outer layers of Drierite (#7779-18-9, Drierite), separated by glass wool (#11-388, Fisher Scientific).

To re-humidify the air to ~9 parts per thousand (ppt) water vapor content, it was then passed through Nafion tubing (#TT-070, Perma Pure, LLC) submerged in deionized water. Bev-A-line nonpermeable tubing (#56280, United States Plastic Corp.) was used to connect all system components.

#### Airflow regulation

Re-humidified, CO_2_-free air was split into two streams, each regulated by mass flow controllers to maintain a constant flow rate of 25 mL/min. One stream provided reference air to the CO_2_ and O_2_ analyzers (controlled by a Side-Trak 840 Series, Sierra Instruments, Inc MFCV), while the second stream provided flow to the fly chambers (controlled by the MAVEn’s internal mass flow-controlled system).

#### Respirometry chambers and MAVEn system’s airflow management

Flies were housed in Sable Systems RC chambers (70 mm × 20 mm borosilicate glass tubes), integrated into a continuous flow through MAVEn system. The MAVEn splits airflow into 16 channels for fly chambers and one dedicated baseline channel. Airflow is constantly and continuously maintained through all fly chambers while the MAVEn’s multiplexing system will switch the ‘active’ chamber’s airflow toward the analyzer chain for a preset dwell time of 120 s per chamber, and 60 s for baseline, with a baseline interleave ratio of 4 to ensure accuracy in O_2_ measurement (i.e. a baseline measurement after every four chambers). In this system, the MAVEn manages airflow to allow for the sequential measurements of individual chamber gas parameters and airflow rates. Experimental respirometry recording was conducted for 24 hr periods. System control and data acquisition were handled via Sable Systems MAVEn Controller software.

#### CO_2_ production measurement

Baseline CO_2_ levels were recorded from the reference air prior to entering the chambers. As flies respired, the CO_2_ released in each chamber was subsequently flushed to the CO_2_ analyzer (Li-7000, LI-COR Biosciences) via the MAVEn system.

#### O_2_ consumption measurement

Similarly, O_2_ consumed by flies was measured immediately using the Oxzilla differential O_2_ analyzer (Oxzilla II Oxygen Analyzer, Sable Systems International) placed downstream from the CO_2_ analyzer in the analyzer chain. In this setup-controlled flow, reference air exiting the CO_2_ analyzer’s reference cell was routed into the Oxzilla’s reference channel, while animal air exiting the CO_2_ analyzer’s sample cell was routed to the Oxzilla’s sample channel. To prevent dilution effects, water vapor was removed prior to O_2_ analysis using small scrubbing columns.

#### Water vapor scrubbing columns

Separate scrubbing columns were used for reference and animal airflows and placed in-line directly prior to the Oxzilla differential O_2_ analyzer’s reference and sample channel inlet ports. Each column was made from a 10 mL syringe body (#14955459, Fisher Scientific) filled with an inner layer of Ascarite and two outer layers of magnesium perchlorate (M54, Fisher Scientific), separated by glass wool. Bev-A-line tubing was secured with rubber stoppers (#14-135E, Fisher Scientific). Columns were replaced twice during each 24 hr experiment to maintain performance.

### Carbon dioxide and oxygen analysis and calculations

O_2_ consumption was quantified by measuring the decrease in O_2_ concentration as air passed through chambers containing flies. To ensure accuracy, a Savitzky-Golay filter (25 s window) was applied to smooth the raw signal, followed by a lag correction of 127 s accounted for delay between chamber exit and O_2_ analyzer input. To match dry atmospheric air, the reference air was baseline-corrected to 20.95% O_2_. For both reference and animal air, the %O_2_ values were converted to O_2_ fractional contents by dividing percentage values by 100 (thus baseline air has a fractional content of 0.2095). The VO_2_ value from an empty chamber was subtracted from experimental values (Lighton, 2008):

$$\displaystyle \rm VO_2 = (Animal\, O_2 - Reference\, O_2) \times Flow\, rate / (1 - Reference\, O_2)$$

VO_2_ values were expressed in μL/hr by summing all 5 min bins into hourly averages.

CO_2_ production was quantified by measuring the increase in CO_2_ concentration as air passed through chambers containing flies. To ensure accuracy, a Savitzky-Golay filter (25 s window) was applied to smooth the raw signal, followed by a lag correction of 22 s with Z correction and 28 s without Z correction accounted for delay between chamber exit and O_2_ analyzer input. To match dry atmospheric air, the reference air was baseline-corrected to 0 parts per million (ppm) CO_2_. For both reference and animal air, the ppm CO_2_ values were converted to CO_2_ fractional contents by dividing percentage values by 1,000,000. The VCO_2_ value from an empty chamber was subtracted from experimental values (Lighton, 2008):

$$\displaystyle \rm VCO_2= (Reference\, CO_2 + Animal\, CO_2) \times Flow\, rate/1-Reference\, CO_2$$

VCO_2_ values were expressed in μL/hr by summing all 5 min bins into hourly averages.

RQ was calculated as:

$$\displaystyle \rm RQ = VCO_2 / VO_2.$$

RQ was used as an index of relative shifts in substrate utilization over time and between genotypes, interpreted conservatively rather than as a precise quantitative measure of absolute carbohydrate versus lipid oxidation, as this has not been directly characterized in flies.

### Gut tissue respirometry

OCR was measured in dissected gut tissue using the Resipher System (Lucid Scientific, GA, USA). Male flies were briefly anesthetized on ice, and whole guts were dissected in Schneider’s Media. 96-Well flat-bottom plates were coated with 50 µL of poly-D-lysine for 30 min to promote tissue adhesion; each well was then filled with 300 µL of Schneider’s Media, a single gut was placed in a fixed position within each well, and the plate was fitted with the Resipher sensor lid. The Resipher hub recorded OCR (fmol/mm^2^/s) continuously for 72 hr in a humidified incubator at 27°C. OCR was extracted in 4 hr bins and averaged over the 24 hr window beginning 12 hr after loading (the 12–36 hr interval, once tissue respiration had stabilized). Experiments were performed in three independent runs; values were pooled, median-normalized to the *iso^31^* controls within each run, and log2(x+10)-transformed. A single *iso^31^* gut in the third run of the *per^01^* experiment was identified as an outlier (Prism ‘Identify Outliers’) and excluded; no other values were removed. Normalized OCR was compared between each mutant and *iso^31^* using the Mann-Whitney test in GraphPad Prism 10, with significance at p<0.05 (*fmn* vs *iso^31^*, *n*=12 vs 12 guts; *per^01^* vs *iso^31^*, *n*=12 vs 11 guts after the single *iso^31^* exclusion).

### Metabolite extraction and LC-MS measurements

The metabolomics dataset analyzed in this study was previously published and is publicly available, as described in detail in Malik et al., 2024; Malik et al., 2018. In the present study, these data were integrated with respirometry measurements to assess temporal relationships between metabolite abundance and respiratory output. Briefly, polar metabolites were extracted from fly bodies sampled every 2 hr from ZT0 to ZT22 using a modified Bligh-Dyer extraction method, as previously reported (Malik et al., 2018; Sengupta and Weljie, 2019). The polar fraction of each extract was dried under vacuum and reconstituted in 100 μL of acetonitrile:Milli-Q water, followed by vortexing for 20 s. Samples were analyzed using an ion-switching LC-MS method, and peak integration and data processing were performed according to previously published procedures (Malik et al., 2024).

### Statistical analysis

Statistical analyses were performed using GraphPad Prism 10 (RRID:SCR_002798) unless otherwise specified. Non-parametric Spearman rank correlation was used to evaluate relationships among metabolic parameters. Metabolites with p-values below 0.05 were considered statistically significant and selected for further investigation. Group comparisons were performed using the statistical tests indicated for each analysis and figure, including Kruskal-Wallis tests followed by Dunn’s multiple-comparisons tests, one-way ANOVA followed by Dunnett’s multiple-comparisons tests, and Mann-Whitney tests for gut tissue OCR.

### Metabolomics-respirometry integration and lag analysis

To integrate respirometry with metabolomics, RQ was recorded continuously at 1 s resolution and averaged into 5 min bins. Because steady-state metabolite measurements were acquired at 2 hr intervals, we extracted the RQ values corresponding to each 2 hr ZT sampling point from the 5 min binned dataset to generate time-matched RQ-metabolite pairs. We additionally evaluated temporal relationships using a lag analysis by systematically shifting the RQ time series relative to the metabolite timepoints (−120, –60, −30, –15, −5, +5, +15, +30, +60, and +120 min). Metabolite abundances were normalized as described above, and associations between RQ and individual metabolites were quantified using Spearman rank correlations (*ρ*) at each lag. Metabolites showing strong associations (e.g. |*ρ*|>0.7 with nominal p<0.05) were carried forward for visualization and summary, and lag direction was interpreted as metabolites preceding (negative lag) or following (positive lag) changes in RQ.

### Rhythmicity analysis

Rhythmicity analysis was performed using Nitecap, a tool for circadian and rhythmic analyses (Brooks et al., 2022). Time-series metabolic data were analyzed to compute rhythmic parameters using the RAIN algorithm (Thaben and Westermark, 2014). Rhythms were assessed for statistical significance using FDR-adjusted p-values, ensuring robust detection while minimizing false positives. The lag parameters of each rhythm were computed using JTK cycle algorithm (Hughes et al., 2010).

### Pathway analysis

Significant metabolites identified from univariate analyses were used for metabolic pathway analysis via MetaboAnalyst (RRID:SCR_015539) 5.0. Metabolites were uploaded using HMDB (RRID:SCR_007712) identifiers and analyzed using the hypergeometric enrichment method, with relative-betweenness centrality applied for topology analysis based on the *D. melanogaster* (KEGG; RRID:SCR_012773) pathway library. Pathways with a p-value less than 0.05 were considered statistically significant.

## Data Availability

All data used to generate figures in this study has been made available as supplemental tables.

## Additional files

Supplementary file 1. Supplementary tables. (A) Metabolites showing strong lag-dependent correlations with respiratory quotient (RQ) across genotypes and lighting conditions, with best lag direction, lag time, Spearman *ρ* at the strongest lag, nominal p-value, and Benjamini-Hochberg FDR. (B) Metabolite set enrichment analysis (MSEA over-representation analysis against KEGG pathways) for each genotype/condition, corresponding to the enrichment panels in Figures 6—8. (C) Experimental unit, sample size, and error representation for the respirometry-based figures (Figures 1—3, Figure 1—figure supplements 1–4).

MDAR checklist
