# Supplementary material for: Integrated respirometry and metabolomics unveil circadian metabolic dynamics in Drosophila
Source: eLife. 2026 Sep 24;14:RP108681. doi: 10.7554/eLife.108681 (PMC13609478; doi:10.7554/eLife.108681)
Supplement: Supplementary file 1 [file elife-108681-mdarchecklist1.docx]

**Materials Design Analysis Reporting (MDAR)**

**Checklist for Authors**

**Manuscript:** Integrated Respirometry and Metabolomics Unveil Circadian Metabolic Dynamics in Drosophila
Authors: Akhtar et al.

| **MDAR item** | **Minimum reporting expectation** | **Where provided / response** | **N/A** |
| --- | --- | --- | --- |
| **Materials:** | | | |
| **Newly created materials** | Disclose availability of newly created materials and any restrictions. | No newly created biological materials (e.g., new strains, cell lines, plasmids, antibodies) are described in this study. | **N/A** |
| **Antibodies** | For commercial antibodies, provide supplier, catalogue number and RRID where available. | No antibodies are used or described. | **N/A** |
| **DNA and RNA sequences** | Provide sequences or repository access for short novel DNA/RNA sequences, primers or probes. | No novel DNA/RNA sequences, primers or probes are used or described. | **N/A** |
| **Cell materials** | For cell lines/primary cultures, report species, strain/source and relevant identifiers. | No cell lines or primary cultures are used. | **N/A** |
| **Experimental animals / model organisms** | Report species, strain, sex, age, genetic modification status and repository/supplier identifier where available. | Methods – Drosophila Strains, Entrainment and Collection. Species: Drosophila melanogaster. Strains: isogenic control (iso/iso31), fumin (fmn; Kume et al., 2005, Ref. 21), sleepless (sss; Koh et al., 2008; Chen et al., 2015; Wu et al., 2010, Refs. 22–24), and period-null (per01; Konopka and Benzer, 1971, Ref. 25). Sex: males only. Age: 5–7 days after eclosion. Housing/diet: standard cornmeal/molasses medium at 25 °C. Lighting: 12 h:12 h LD (ZT0 = lights-on; ZT12 = lights-off); WT also tested after ≥24 h in constant darkness (WT-DD). Stock/repository identifiers are not listed in the manuscript. |  |
| **Plants and microbes** | Report species/strain/source for plants or microbes studied. | No plants or microbial strains are experimental subjects. Background microbial metabolism is controlled in empty-food respirometry chambers, but microbes are not experimentally characterized. | **N/A** |
| **Human research participants** | Where applicable, report participant demographics within privacy constraints. | No human participants are included in this study. | **N/A** |
| **Design:** | | | |
| **Study protocol** | If the study was preregistered, provide registration/DOI; clinical trials require registration details. | No preregistered study protocol or clinical trial is described. | **N/A** |
| **Laboratory protocol** | Provide DOI/citation if detailed step-by-step protocols are available. | Methods provide detailed respirometry and gut-OCR procedures. Previously established methods are cited for metabolic-rate calculations and metabolomics (including refs. 27, 30 and 31). |  |
| **Sample size determination** | State whether sample size was determined prospectively; if no power analysis was used, explain how replicate number was chosen. | No formal power analysis was performed. Replicate number was based on the capacity of the MAVEn flow-through respirometry system (16 fly chambers) together with empirical optimization of group size; 25 flies per chamber produced a clear, reliable respiratory signal. Four chambers were used per genotype/condition in each run, and recordings were repeated across three independent experimental days, giving n = 12 chambers per genotype/condition, with chamber as the experimental unit (Methods – Respirometry Setup; Supplementary Table 3). Gut OCR used three independent runs with individual guts as the experimental unit (typically n = 12 per genotype; Methods – Gut Tissue Respirometry). |  |
| **Randomisation** | State whether and how samples/experimental groups were randomized; if not done, state 'not done'. | Not done. Experimental groups were genetically defined genotypes/conditions rather than randomly allocated treatments. Four chambers per genotype/condition were assayed within MAVEn runs. |  |
| **Blinding / masking** | State whether and how blinding was used during allocation, data collection or analysis; if not done, state 'not done'. | Not done. Genotype identity was known during chamber loading and analysis. Respirometry (VO₂, VCO₂, RQ) and gut OCR were acquired instrumentally rather than by subjective scoring. |  |
| **Inclusion/exclusion criteria** | State inclusion/exclusion criteria and whether they were prespecified. | Inclusion: male flies, 5–7 days old, 25 flies per respirometry chamber after optimization of group size for a clear respiratory signal (Methods – Drosophila Strains; Respirometry Setup). Whole-fly respirometry included food-only control chambers to subtract background microbial metabolism. Gut OCR inclusion: dissected male guts with OCR averaged over the 12–36 h window after loading, once tissue respiration had stabilized. Exclusion: one iso31 gut in the third per01 run was identified as an outlier using GraphPad Prism “Identify Outliers” and excluded; no other gut OCR values were removed (Methods – Gut Tissue Respirometry; Figure 9 legend). No other samples were excluded from the respirometry analyses. |  |
| **Sample definition and in-laboratory replication** | Report number of independent repetitions and define the experimental unit / biological versus technical replication. | Methods – Respirometry Setup and Supplementary Table 3: chamber is the experimental unit; 25 flies/chamber, 4 chambers/experiment, 3 experimental days, typically n = 12 chambers/genotype/condition. Methods – Gut Tissue Respirometry: three independent runs; individual guts analyzed (fmn vs iso31, n = 12 vs 12; per01 vs iso31, n = 12 vs 11 after one exclusion). Figure legends report n and SEM. The reused metabolomics dataset is described in Methods and refs. 27/31. |  |
| **Ethics** | For human/animal/specimen studies, report approving authority and approval number where applicable. | N/A. This study uses Drosophila melanogaster (an invertebrate) and includes no human participants or vertebrate animals. Institutional animal-ethics (IACUC) approval is not required for this work. | **N/A** |
| **Dual Use Research of Concern (DURC)** | If subject to DURC regulations, report approval authority and reference number. | No dual-use research of concern is apparent from the study design. | **N/A** |
| **Analysis:** | | | |
| **Attrition / omitted data** | Report omitted samples/data points and justification, including whether criteria were pre-established. | Methods – Gut Tissue Respirometry and Figure 9 legend: one iso31 gut in the third per01 run was excluded after identification as an outlier in Prism; no other gut OCR values were removed. Correlation plots use rank-based visualization to reduce outlier influence, but the underlying correlation selection criteria are stated. |  |
| **Statistics** | Describe and justify statistical tests; report n, center/dispersion, multiple-testing correction and exact p-values where appropriate. | Methods – Statistical Analysis, Metabolomics-respirometry integration and lag analysis, Rhythmicity analysis, Pathway Analysis, and Gut Tissue Respirometry; figure legends and tables. Analyses include Spearman correlations, genotype comparisons, Mann–Whitney testing for gut OCR, RAIN/JTK_CYCLE rhythmicity analysis with FDR adjustment, and pathway enrichment. Figure legends/Supplementary Table 3 report experimental units, n and SEM. GraphPad Prism 10 (RRID:SCR_002798) was used for applicable statistical analyses. |  |
| **Data availability** | Provide access details for newly created and reused datasets, including repository identifiers/DOIs/URLs where available. | All data used to generate the figures are provided with the article as supplemental/source-data tables. The metabolomics dataset analyzed here was previously published and is publicly available (Methods – Metabolite Extraction and LCMS Measurements; refs. 27 and 31). |  |
| **Code availability** | Provide access details for code/software essential to reproduce the main findings; newly generated code should be deposited where possible. | No newly generated custom analysis code is described. Analyses used published/commercial software: Sable Systems MAVEn Controller; GraphPad Prism 10 (RRID:SCR_002798); Nitecap with RAIN and JTK_CYCLE (Brooks et al., 2022; Thaben and Westermark, 2014; Hughes et al., 2010); and MetaboAnalyst 5.0 (RRID:SCR_015539). | **N/A** |
| **Reporting:** | | | |
| **Adherence to community standards** | State whether relevant reporting standards/guidelines (e.g., ARRIVE, STRANGE or other discipline-specific guidance) were followed and whether a checklist is supplied. | This MDAR checklist is supplied with the submission. The manuscript reports species, strain, sex, age, lighting/entrainment, temperature, diet, experimental unit, sample size, and the limitation of using males only (Methods; Discussion). No additional reporting checklist is cited. |  |
