# Supplementary material for: Integrated respirometry and metabolomics unveil circadian metabolic dynamics in Drosophila
Source: eLife. 2026 Sep 24;14:RP108681. doi: 10.7554/eLife.108681 (PMC13609478; doi:10.7554/eLife.108681)
Supplement: Supplementary file 2 [file elife-108681-supp1.docx]

**Supplementary File 1**

Supplementary tables A–C supporting Akhtar et al., Integrated Respirometry and Metabolomics Unveil Circadian Metabolic Dynamics in Drosophila.

**Table A.** **Metabolites showing strong lag-dependent correlations with respiratory quotient (RQ) across genotypes and lighting conditions.**
Note: Metabolites were included if they showed a strong metabolite–RQ association at one or more temporal lags, defined as |ρ| ≥ 0.7 with nominal p < 0.05. Nominal p-values are reported at the strongest lag for each metabolite. Benjamini–Hochberg false discovery rate (BH-FDR) adjusted p-values were calculated from the full metabolite × lag nominal p-value set within each genotype/condition, including all metabolites and all lag time points tested, and are reported at the strongest lag. BH-FDR adjusted p < 0.05 was considered significant after multiple-testing correction.

| Genotype/ condition | Figure | Metabolite | Best lag direction | Best lag time | Spearman ρ at strongest lag | Nominal  p-value |
| --- | --- | --- | --- | --- | --- | --- |
| WT-LD | Fig. 6 | Quinolinate | Negative lag | −120 min | −0.77 | 0.0049 |
| WT-LD | Fig. 6 | Hydroxyhexadecenoylcarnitine | Positive lag | +120 min | +0.78 | 0.0038 |
| WT-LD | Fig. 6 | NADP⁺ | Positive lag | +120 min | +0.75 | 0.007 |
| WT-LD | Fig. 6 | FADH | Positive lag | +120 min | +0.76 | 0.0062 |
| WT-LD | Fig. 6 | LPC 20:5 | Positive lag | +120 min | +0.76 | 0.0062 |
| WT-LD | Fig. 6 | Nicotinamide riboside | Positive lag | +120 min | +0.75 | 0.007 |
| WT-LD | Fig. 6 | Glucose | Positive lag | +120 min | +0.71 | 0.0129 |
| *fmn* | Fig. 7 | Glutamine | Positive lag | +30 min | +0.71 | 0.0129 |
| *fmn* | Fig. 7 | Methylhistidines | Positive lag | +30 min | +0.90 | 0.0002 |
| *fmn* | Fig. 7 | Inosine | Positive lag | +30 min | +0.85 | 0.0009 |
| *fmn* | Fig. 7 | Citrulline | Positive lag | +120 min | +0.84 | 0.0011 |
| *fmn* | Fig. 7 | LPC 18:0 | Positive lag | +60 min | +0.71 | 0.0129 |
| *fmn* | Fig. 7 | LPC 20:1 | Negative lag | −15 min | −0.71 | 0.0129 |
| *sss* | Fig. 7 | Monosaccharide | Negative lag | −5 min | −0.76 | 0.0055 |
| *sss* | Fig. 7 | 4-Guanidinobutanoic acid | Negative lag | −60 min | −0.73 | 0.0087 |
| *sss* | Fig. 7 | Biotin | Negative lag | −60 min | −0.71 | 0.0118 |
| *sss* | Fig. 7 | Citrulline | Negative lag | −120 min | −0.74 | 0.0078 |
| *sss* | Fig. 7 | Sucrose | Negative lag | −120 min | −0.83 | 0.0013 |
| *sss* | Fig. 7 | Dimethylglycine/2Aminobutyrate | Positive lag | +60 min | +0.73 | 0.0096 |
| *sss* | Fig. 7 | Glycine | Positive lag | +60 min | +0.73 | 0.0087 |
| *sss* | Fig. 7 | Stearoylcarnitine | Positive lag | +30 min | +0.75 | 0.007 |
| *per^01^* | Fig. 8 | Creatine | Positive lag | +120 min | +0.73 | 0.0096 |
| *per^01^* | Fig. 8 | Glutamine | Negative lag | −60 min | −0.72 | 0.0106 |
| *per^01^* | Fig. 8 | Spermidine | Negative lag | −30 min | −0.78 | 0.0043 |
| *per^01^* | Fig. 8 | dAMP | Negative lag | −30 min | −0.83 | 0.0016 |
| *per^01^* | Fig. 8 | Lauroylcarnitine | Negative lag | −30 min | −0.72 | 0.0106 |
| *per^01^* | Fig. 8 | Methylhistidines | Negative lag | −30 min | −0.71 | 0.0129 |
| *per^01^* | Fig. 8 | Creatinine | Positive lag | +120 min | +0.76 | 0.0055 |
| *per^01^* | Fig. 8 | NADPH | Positive lag | +120 min | +0.83 | 0.0013 |
| *per^01^* | Fig. 8 | Adenosine | Negative lag | −120 min | −0.73 | 0.0096 |
| *per^01^* | Fig. 8 | LPC 14:0 | Negative lag | −15 min | −0.81 | 0.0022 |
| *per^01^* | Fig. 8 | Acetylputrescine | Negative lag | −30 min | −0.84 | 0.0011 |
| *per^01^* | Fig. 8 | Cis-5-Tetradecenoylcarnitine | Negative lag | −30 min | −0.78 | 0.0038 |
| *per^01^* | Fig. 8 | ADP-ribose | Negative lag | −5 min | −0.74 | 0.0078 |
| *per^01^* | Fig. 8 | Alpha-ketoglutarate | Positive lag | +5 min | +0.77 | 0.0049 |
| WT-DD | Fig. 8 | Phosphoserine | Positive lag | +120 min | +0.78 | 0.0038 |
| WT-DD | Fig. 8 | Pyruvate | Negative lag | −60 min | −0.72 | 0.0106 |
| WT-DD | Fig. 8 | Stearoylcarnitine | Negative lag | −5 min | −0.71 | 0.0118 |
| WT-DD | Fig. 8 | 3-Indoleacetic acid | Negative lag | −60 min | −0.71 | 0.0129 |

**Lag interpretation:** Negative lag indicates that metabolite changes precede RQ changes and positive lag indicates that metabolite changes follow RQ changes. Metabolites correspond to those shown in the metabolite-RQ correlation heatmaps in Figures 6-8, with nominal p-values and BH-FDR values calculated from the full correlation output.

**Table B. Metabolite Set Enrichment Analysis (MSEA ORA)**

| Genotype / condition | Figure | KEGG pathway | Total metabolites in set | Expected hits | Observed hits | Enrichment ratio | Raw  p-value | Holm-adjusted  p-value | FDR | Contributing metabolites |
| --- | --- | --- | --- | --- | --- | --- | --- | --- | --- | --- |
| WT-LD | **6** | **Nicotinate and nicotinamide metabolism** | **15** | **0.088** | **3** | **34.21** | **6.08e-05** | **0.0049** | **0.0049** | **L-Aspartic acid, Quinolinic acid, Nicotinamide riboside** |
| WT-LD | **6** | **Alanine, aspartate and glutamate metabolism** | **28** | **0.164** | **3** | **18.29** | **0.0004** | **0.0333** | **0.0169** | **L-Aspartic acid, Citric acid, Carbamoyl phosphate** |
| WT-LD | **6** | **Arginine biosynthesis** | **14** | **0.082** | **2** | **24.42** | **0.0027** | **0.208** | **0.0712** | **L-Aspartic acid, Carbamoyl phosphate** |
| WT-LD | **6** | **Citrate cycle (TCA cycle)** | **20** | **0.117** | **2** | **17.09** | **0.0055** | **0.421** | **0.109** | **Malic acid, Citric acid** |
| WT-LD | **6** | **Neomycin, kanamycin and gentamicin biosynthesis** | **2** | **0.012** | **1** | **85.47** | **0.0117** | **0.887** | **0.173** | **D-Glucose** |
| WT-LD | **6** | **Glyoxylate and dicarboxylate metabolism** | **31** | **0.181** | **2** | **11.05** | **0.0129** | **0.971** | **0.173** | **Citric acid, Malic acid** |
| WT-LD | **6** | **Nitrogen metabolism** | **6** | **0.035** | **1** | **28.49** | **0.0346** | **1** | **0.396** | **Carbamoyl phosphate** |
| WT-LD | 6 | Histidine metabolism | 16 | 0.094 | 1 | 10.68 | 0.09 | 1 | 0.847 |  |
| WT-LD | 6 | Starch and sucrose metabolism | 18 | 0.105 | 1 | 9.52 | 0.101 | 1 | 0.847 |  |
| WT-LD | 6 | Pantothenate and CoA biosynthesis | 20 | 0.117 | 1 | 8.55 | 0.111 | 1 | 0.847 |  |
| WT-LD | 6 | beta-Alanine metabolism | 21 | 0.123 | 1 | 8.13 | 0.117 | 1 | 0.847 |  |
| WT-LD | 6 | Pyruvate metabolism | 23 | 0.135 | 1 | 7.41 | 0.127 | 1 | 0.847 |  |
| WT-LD | 6 | Galactose metabolism | 27 | 0.158 | 1 | 6.33 | 0.148 | 1 | 0.908 |  |
| WT-LD | 6 | Glycine, serine and threonine metabolism | 33 | 0.193 | 1 | 5.18 | 0.178 | 1 | 0.947 |  |
| WT-LD | 6 | Cysteine and methionine metabolism | 33 | 0.193 | 1 | 5.18 | 0.178 | 1 | 0.947 |  |
| WT-LD | 6 | Pyrimidine metabolism | 39 | 0.228 | 1 | 4.39 | 0.207 | 1 | 1 |  |
| *fmn* | **7** | **Arginine biosynthesis** | **14** | **0.046** | **2** | **43.96** | **0.0008** | **0.0606** | **0.0606** | **Citrulline,**  **Glutamine** |
| *fmn* | **7** | **Purine metabolism** | **70** | **0.227** | **2** | **8.81** | **0.0187** | **1** | **0.516** | **Glutamine, Inosine** |
| *fmn* | **7** | **Nitrogen metabolism** | **6** | **0.02** | **1** | **51.28** | **0.0194** | **1** | **0.516** | **Glutamine** |
| *fmn* | **7** | **Butanoate metabolism** | **15** | **0.049** | **1** | **20.53** | **0.0479** | **1** | **0.816** | **2-Hydroxyglutarate** |
| *fmn* | **7** | **Histidine metabolism** | **16** | **0.052** | **1** | **19.23** | **0.051** | **1** | **0.816** | **Methylhistidine** |
| *fmn* | 7 | Alanine, aspartate and glutamate metabolism | 28 | 0.091 | 1 | 10.99 | 0.0878 | 1 | 1 |  |
| *fmn* | 7 | Glyoxylate and dicarboxylate metabolism | 31 | 0.101 | 1 | 9.9 | 0.0969 | 1 | 1 |  |
| *fmn* | 7 | Pyrimidine metabolism | 39 | 0.127 | 1 | 7.87 | 0.121 | 1 | 1 |  |
| *sss* | **7** | **Glycine, serine and threonine metabolism** | **33** | **0.214** | **3** | **14.02** | **0.001** | **0.0779** | **0.0779** | **Choline, Dimethylglycine, Glycine** |
| *sss* | **7** | **Butanoate metabolism** | **15** | **0.098** | **2** | **20.51** | **0.0038** | **0.301** | **0.147** | **Acetoacetic acid, Succinic acid** |
| *sss* | **7** | **Starch and sucrose metabolism** | **18** | **0.117** | **2** | **17.09** | **0.0055** | **0.429** | **0.147** | **Sucrose, D-Glucose** |
| *sss* | **7** | **Galactose metabolism** | **27** | **0.175** | **2** | **11.43** | **0.0122** | **0.942** | **0.207** | **Sucrose, D-Glucose** |
| *sss* | **7** | **Neomycin, kanamycin and gentamicin biosynthesis** | **2** | **0.013** | **1** | **76.92** | **0.013** | **0.985** | **0.207** | **D-Glucose** |
| *sss* | 7 | Biotin metabolism | 10 | 0.065 | 1 | 15.38 | 0.0633 | 1 | 0.844 |  |
| *sss* | 7 | Arginine biosynthesis | 14 | 0.091 | 1 | 10.99 | 0.0876 | 1 | 1 |  |
| *sss* | 7 | Citrate cycle (TCA cycle) | 20 | 0.13 | 1 | 7.69 | 0.123 | 1 | 1 |  |
| *sss* | 7 | Propanoate metabolism | 21 | 0.136 | 1 | 7.35 | 0.129 | 1 | 1 |  |
| *sss* | 7 | Alanine, aspartate and glutamate metabolism | 28 | 0.182 | 1 | 5.49 | 0.168 | 1 | 1 |  |
| *sss* | 7 | Glutathione metabolism | 28 | 0.182 | 1 | 5.49 | 0.168 | 1 | 1 |  |
| *sss* | 7 | Lipoic acid metabolism | 28 | 0.182 | 1 | 5.49 | 0.168 | 1 | 1 |  |
| *sss* | 7 | Glyoxylate and dicarboxylate metabolism | 31 | 0.201 | 1 | 4.98 | 0.185 | 1 | 1 |  |
| *sss* | 7 | Porphyrin metabolism | 31 | 0.201 | 1 | 4.98 | 0.185 | 1 | 1 |  |
| *sss* | 7 | Arginine and proline metabolism | 36 | 0.234 | 1 | 4.27 | 0.211 | 1 | 1 |  |
| *sss* | 7 | Glycerophospholipid metabolism | 36 | 0.234 | 1 | 4.27 | 0.211 | 1 | 1 |  |
| *sss* | 7 | Valine, leucine and isoleucine degradation | 39 | 0.253 | 1 | 3.95 | 0.227 | 1 | 1 |  |
| *sss* | 7 | Tyrosine metabolism | 42 | 0.273 | 1 | 3.66 | 0.242 | 1 | 1 |  |
| *sss* | 7 | Primary bile acid biosynthesis | 46 | 0.299 | 1 | 3.34 | 0.262 | 1 | 1 |  |
| *per^01^* | **8** | **Alanine, aspartate and glutamate metabolism** | **28** | **0.4** | **4** | **10.0** | **0.0005** | **0.041** | **0.0334** | **L-Alanine, Glutamine, Fumaric acid, Oxoglutaric acid** |
| *per^01^* | **8** | **Arginine biosynthesis** | **14** | **0.2** | **3** | **15.0** | **0.0008** | **0.0659** | **0.0334** | **Glutamine, Oxoglutaric acid, Fumaric acid** |
| *per^01^* | **8** | **Arginine and proline metabolism** | **36** | **0.515** | **4** | **7.77** | **0.0014** | **0.107** | **0.0365** | **Creatine, Spermidine, N-Acetylputrescine, 4-Hydroxyproline** |
| *per^01^* | **8** | **Purine metabolism** | **70** | **1.0** | **5** | **5.0** | **0.0024** | **0.187** | **0.0485** | **Glutamine, Adenosine, Deoxyadenosine monophosphate, Xanthosine, Adenosine diphosphate ribose** |
| *per^01^* | **8** | **Nicotinate and nicotinamide metabolism** | **15** | **0.214** | **2** | **9.35** | **0.0183** | **1** | **0.293** | **Quinolinic acid, Niacinamide** |
| *per^01^* | **8** | **Citrate cycle (TCA cycle)** | **20** | **0.286** | **2** | **6.99** | **0.0317** | **1** | **0.423** | **Oxoglutaric acid, Fumaric acid** |
| *per^01^* | **8** | **Glutathione metabolism** | **28** | **0.4** | **2** | **5.0** | **0.0589** | **1** | **0.674** | **NADPH, Spermidine** |
| *per^01^* | 8 | Cysteine and methionine metabolism | 33 | 0.472 | 2 | 4.24 | 0.0789 | 1 | 0.737 |  |
| *per^01^* | 8 | Nitrogen metabolism | 6 | 0.086 | 1 | 11.66 | 0.0829 | 1 | 0.737 |  |
| *per^01^* | 8 | Pyrimidine metabolism | 39 | 0.558 | 2 | 3.58 | 0.105 | 1 | 0.75 |  |
| *per^01^* | 8 | Valine, leucine and isoleucine biosynthesis | 8 | 0.114 | 1 | 8.77 | 0.109 | 1 | 0.75 |  |
| *per^01^* | 8 | Tyrosine metabolism | 42 | 0.6 | 2 | 3.33 | 0.119 | 1 | 0.75 |  |
| *per^01^* | 8 | Ascorbate and aldarate metabolism | 9 | 0.129 | 1 | 7.75 | 0.122 | 1 | 0.75 |  |
| *per^01^* | 8 | Butanoate metabolism | 15 | 0.214 | 1 | 4.67 | 0.195 | 1 | 1 |  |
| *per^01^* | 8 | Histidine metabolism | 16 | 0.229 | 1 | 4.37 | 0.207 | 1 | 1 |  |
| *per^01^* | 8 | Selenocompound metabolism | 20 | 0.286 | 1 | 3.5 | 0.252 | 1 | 1 |  |
| *per^01^* | 8 | beta-Alanine metabolism | 21 | 0.3 | 1 | 3.33 | 0.262 | 1 | 1 |  |
| *per^01^* | 8 | Pyruvate metabolism | 23 | 0.329 | 1 | 3.04 | 0.284 | 1 | 1 |  |
| *per^01^* | 8 | Galactose metabolism | 27 | 0.386 | 1 | 2.59 | 0.324 | 1 | 1 |  |
| *per^01^* | 8 | Lipoic acid metabolism | 28 | 0.4 | 1 | 2.5 | 0.334 | 1 | 1 |  |
| *per^01^* | 8 | Inositol phosphate metabolism | 30 | 0.429 | 1 | 2.33 | 0.353 | 1 | 1 |  |
| *per^01^* | 8 | Glyoxylate and dicarboxylate metabolism | 31 | 0.443 | 1 | 2.26 | 0.363 | 1 | 1 |  |
| *per^01^* | 8 | Glycine, serine and threonine metabolism | 33 | 0.472 | 1 | 2.12 | 0.381 | 1 | 1 |  |
| *per^01^* | 8 | Valine, leucine and isoleucine degradation | 39 | 0.558 | 1 | 1.79 | 0.434 | 1 | 1 |  |
| WT-DD | **8** | **Arginine biosynthesis** | **14** | **0.091** | **3** | **32.97** | **6.94e-05** | **0.0056** | **0.0056** | **Citrulline, Ornithine, Urea** |
| WT-DD | **8** | **Glycine, serine and threonine metabolism** | **33** | **0.214** | **3** | **14.02** | **0.001** | **0.0769** | **0.026** | **L-Cystathionine, Phosphoserine, Pyruvic acid** |
| WT-DD | **8** | **Cysteine and methionine metabolism** | **33** | **0.214** | **3** | **14.02** | **0.001** | **0.0769** | **0.026** | **L-Cystathionine, Phosphoserine, Pyruvic acid** |
| WT-DD | **8** | **Arginine and proline metabolism** | **36** | **0.234** | **2** | **8.55** | **0.0213** | **1** | **0.426** | **Ornithine, Pyruvic acid** |
| WT-DD | 8 | Butanoate metabolism | 15 | 0.098 | 1 | 10.26 | 0.0936 | 1 | 1 |  |
| WT-DD | 8 | Starch and sucrose metabolism | 18 | 0.117 | 1 | 8.55 | 0.111 | 1 | 1 |  |
| WT-DD | 8 | Citrate cycle (TCA cycle) | 20 | 0.13 | 1 | 7.69 | 0.123 | 1 | 1 |  |
| WT-DD | 8 | Pyruvate metabolism | 23 | 0.149 | 1 | 6.71 | 0.14 | 1 | 1 |  |
| WT-DD | 8 | Glycolysis / Gluconeogenesis | 26 | 0.169 | 1 | 5.92 | 0.157 | 1 | 1 |  |
| WT-DD | 8 | Galactose metabolism | 27 | 0.175 | 1 | 5.71 | 0.163 | 1 | 1 |  |
| WT-DD | 8 | Alanine, aspartate and glutamate metabolism | 28 | 0.182 | 1 | 5.49 | 0.168 | 1 | 1 |  |
| WT-DD | 8 | Glutathione metabolism | 28 | 0.182 | 1 | 5.49 | 0.168 | 1 | 1 |  |
| WT-DD | 8 | Lipoic acid metabolism | 28 | 0.182 | 1 | 5.49 | 0.168 | 1 | 1 |  |
| WT-DD | 8 | Glyoxylate and dicarboxylate metabolism | 31 | 0.201 | 1 | 4.98 | 0.185 | 1 | 1 |  |
| WT-DD | 8 | Pyrimidine metabolism | 39 | 0.253 | 1 | 3.95 | 0.227 | 1 | 1 |  |
| WT-DD | 8 | Tryptophan metabolism | 41 | 0.266 | 1 | 3.76 | 0.237 | 1 | 1 |  |
| WT-DD | 8 | Tyrosine metabolism | 42 | 0.273 | 1 | 3.66 | 0.242 | 1 | 1 |  |
| WT-DD | 8 | Purine metabolism | 70 | 0.455 | 1 | 2.2 | 0.373 | 1 | 1 |  |

**Note: MSEA over-representation analysis (ORA) was performed against KEGG pathways for each genotype/condition. Pathways listed correspond to the enrichment panels shown in Figures 6-8. Raw p-values, Holm-adjusted p-values, and FDR values are reported for each pathway. Contributing metabolites are shown for pathways/metabolite sets meeting the nominal significance threshold of p < 0.05. FDR-adjusted significance should be interpreted separately from the nominal p-value threshold.**

**Table C. Experimental unit, sample size, and error representation for respirometry-based figures.**

This table summarizes the experimental unit, chamber-level sample size, and error representation for all respirometry-based analyses shown in Figures 1-3 and Figure 1—figure supplements 1–4. For all respirometry analyses, the chamber was used as the experimental unit. Each chamber contained 25 flies, and each genotype/condition was measured across four chambers per experiment over three experimental days, corresponding to n = 12 chambers per genotype/condition unless otherwise indicated.

| Figure/panel | Data shown | Genotypes  /Conditions | Experimental unit | n per genotype/condition | Error bars/shaded bands |
| --- | --- | --- | --- | --- | --- |
| Fig. 1a-c | 5-min VCO₂, VO₂, RQ traces | WT-LD, *fmn*, *sss*, *per^01^*, WT-DD | Chamber | n = 12 chambers per genotype | Mean ± SEM |
| Fig. 1d-f | 24-h average VCO₂, VO₂, RQ | WT-LD, *fmn*, *sss*, *per^01^*, WT-DD | Chamber | n = 12 chambers per genotype | Mean ± SEM or boxplot details |
| Fig. 1g-i | Body weight / normalized VCO₂, VO₂ | WT, *fmn*, *sss*, *per^01^* | Chamber | n = 12 chambers per genotype | Mean ± SEM |
| Fig. 2 | Rhythmicity time course | WT-LD, *fmn*, *sss*, *per^01^*, WT-DD | Chamber | n = 12 chambers per genotype | Mean ± SEM |
| Fig. 3 | Phase/peak timing | WT-LD, *fmn*, *sss*, *per^01^*, WT-DD | Chamber | n = 12 chambers per genotype | Not applicable / phase estimate |
| Figure 1—figure supplements 1–3 | Individual VCO₂, VO₂, RQ traces | WT-LD, *fmn*, *sss*, *per^01^*, WT-DD | Chamber | 12 chambers per genotype | Mean ± SEM |
| Figure 1—figure supplement 4 | Day vs night averages | WT-LD, *fmn*, *sss*, *per^01^*, WT-DD | Chamber | 12 chambers per genotype | Mean ± SEM or boxplot details |

**Note: Chamber was used as the experimental unit for all respirometry analyses. Each chamber contained 25 flies, and each genotype/condition was measured across four chambers per experiment over three experimental days, corresponding to n = 12 chambers per genotype/condition unless otherwise indicated. Error bars or shaded bands represent SEM. For boxplots, the plotted distribution/error representation is indicated in the corresponding figure legend.**
