# Supplementary material for: Integrated respirometry and metabolomics unveil circadian metabolic dynamics in Drosophila
Source: eLife. 2026 Sep 24;14:RP108681. doi: 10.7554/eLife.108681 (PMC13609478; doi:10.7554/eLife.108681)
Supplement: Peer review history

# Editor report: eLife Assessment

**DOI:** 10.7554/eLife.108681.3.sa0

**Authors:** P Darrell Neufer

**Affiliations:** Wake Forest University School of Medicine United States

**Keywords:** Convincing, Important

This **important** study investigates how sleep loss and circadian disruption affect whole-organ metabolism in flies (*Drosophila melanogaster*) and reports that wild-type flies align metabolism in anticipation of diurnal rhythm, while mutant flies with impaired sleep or circadian function shift to reactive or misaligned metabolism. The integration of chamber-based flow-through respirometry with LC-MS metabolomics is innovative, and the significance of the findings is significant. The strength of evidence needed to support the conclusions is generally **convincing**, although the absence of direct measures of locomotor activity makes it difficult to separate intrinsic metabolic changes from potential behavioral differences in mutants.

# Referee report: Reviewer #1 (Public review)

**DOI:** 10.7554/eLife.108681.3.sa1

Summary:

This study by Akhtar et al. aims to investigate the link between systemic metabolism and respiratory demands, and how sleep and circadian clock regulate metabolic states and respiratory dynamics. The authors leverage genetic mutants that are defective in sleep and circadian behavior in combination with indirect respirometry and steady-state LC-MS-based metabolomics to address this question in the Drosophila model.

First, the authors performed respirometry (on groups of 25 flies) to measure oxygen consumption (VO2) and carbon dioxide production (VCO2) to calculate the respiratory quotient (RQ) across the 24-hour day (12h:12h light-dark cycle) and assess metabolic fuel utilization. They observed that among all the genotypes tested, wild type (WT) flies and per0 flies in LD and WT flies in DD exhibit RQ >1. They concluded the >1 RQ is consistent with active lipogenesis. In contrast, the short-sleep mutants fumin (fmn) and sleepless (sss) showed significantly different RQ; the fmn exhibits a slight reduction in RQ values, suggesting increased reliance on carbohydrate metabolism, while sss exhibit even lower RQ (0.94), consistent with a shift toward lipid and protein catabolism.

The authors then proceeded to bin these measurements in 12-hour partitions, ZT0-12 and ZT12-24, to assess diurnal differences in average values of VO2, VCO2, and RQ. They observed significant day-night differences in metabolic rates in WT-LD flies, with higher rates during the day. The diurnal differences remain in the short-sleep mutants, but the overall metabolic rates are higher. WT-DD flies exhibit the lowest respiratory activity although the day-night differences remain in free-running conditions. Finally, per01 mutants exhibit no significant change in day-night respiratory rates, suggesting that a functional circadian clock is necessary for diurnal differences in metabolic rates.

They then performed finer resolution 24-hours rhythmic analysis (RAIN and JTK) to determine if VO2, VCO2, and RQ exhibit 24-hour rhythmic and if there are genotype-specific differences. Based on their criteria, VCO2 is rhythmic in all conditions tested while VO2 is rhythmic in all conditions except in fmn-LD. Finally, RQ is rhythmic in all 3 mutants but not in WT-LD and WT-DD. Peak phases for the rhythms were deduced using JTK lag values.

The authors proceeded to leverage a previously published steady-state metabolite datasets to investigate potential association of RQ with metabolite profiles. Spearman correlation was performed to identify metabolites that exhibit coupling to respiratory output. Positive and negative lag analysis were subsequently performed to further characterize these associations based on the timing of the metabolite peak changes relative to RQ fluctuations. The authors suggest that a positive lag indicates that metabolite changes occur after shifts in RQ, and a negative lag signifies that metabolite changes precede RQ changes. To visualize metabolic pathways that exhibit these temporal relationships, clustered heatmap and enrichment analysis were performed. Through these analyses, they concluded that both sleep and circadian systems are essential for aligning metabolic substrate selection with energy demands, and different metabolic pathways are misregulated in the different mutants with sleep and circadian defects.

Strength:

The research questions this study explore are significant given metabolism and respiratory demand are central to animal biology. The experimental methods used, including the well characterized fly genetic mutants, the newly developed method for indirect calorimetry measurements, and LC-MS based metabolomics, are all appropriate. This study provides insights into the impact of sleep and circadian rhythm disruption on metabolism and respiratory demand and serves as a foundation for future mechanistic investigations.

Comments on revised version.

The authors have thoughtfully revised the manuscript. They have now provided clarifications regarding perceived conceptual flaws in the original version. They have also performed additional data analysis to support their conclusions and provide clarifications on statistical methods when appropriate. Overall, the revised manuscript is much improved and the conclusions are generally well supported by their results.

# Referee report: Reviewer #2 (Public review)

**DOI:** 10.7554/eLife.108681.3.sa2

This is an innovative and technically strong study that integrates dual-gas respirometry with LC-MS metabolomics to examine how sleep and circadian disruption shape metabolism in Drosophila. The combination of continuous O₂/CO₂ measurements with high-temporal-resolution metabolite profiling is novel and provides fresh insight into how wild-type flies maintain anticipatory fuel alignment, while mutants shift to reactive or misaligned metabolism. The use of lag-shift correlation analysis is particularly clever, as it highlights temporal coordination rather than static associations. Together, the findings advance our understanding of how circadian clocks and sleep contribute to metabolic efficiency and redox balance.

However, there are several areas where the manuscript could be strengthened. The authors should acknowledge that their findings may be gene-specific. Because sleep deprivation was not performed, it remains uncertain whether the observed metabolic shifts generalize to sleep loss broadly or are restricted to the fmn and sss mutants. This concern also connects to the finding of metabolic misalignment under constant darkness despite an intact clock. The conclusion that external entrainment is essential for maintaining energy homeostasis in flies may not translate to mammals. It would help to reference supporting data for the finding and discuss differences across species. Ideally, complementary circadian (light-dark cycle disruption) or sleep deprivation (for several hours) experiments, or citation of comparable studies, would strengthen the generality of the findings. Figures 1-4 are straightforward and clear, but when the manuscript transitions to the metabolite-respiration correlations, there is little description of the metabolomics methods or datasets, which should be clarified. The Discussion is at times repetitive and could be tightened, with the main message (i.e., wild-type flies align metabolism in advance, while mutants do not) kept front and center. Terms such as "anticipatory" and "reactive" should be defined early and used consistently throughout.

Overall, this is a strong and novel contribution. With clarification of scope, refinement of presentation, and a more focused Discussion, the paper will make a significant impact.

Comments on revised version.

The authors have satisfactorily addressed my concerns in the revised manuscript

# Referee report: Reviewer #3 (Public review)

**DOI:** 10.7554/eLife.108681.3.sa3

Summary:

The authors investigate how sleep loss and circadian disruption affect whole-organism metabolism in *Drosophila melanogaster*. They used chamber-based flow-through respirometry to measure oxygen consumption, carbon dioxide production, in wild-type flies and in mutants with impaired sleep or circadian function. These measurements were then integrated with a previously published metabolomics dataset to explore how respiratory dynamics align with metabolic pathways. The central claim is that wild-type flies display anticipatory coordination of metabolic processes with circadian time, while mutants exhibit reactive shifts in substrate use, redox imbalance, and signs of mitochondrial stress.

Strengths:

The study has several strengths. Continuous high-resolution respirometry in flies is challenging, and its application across multiple genotypes provides good comparative insight. The conceptual framework distinguishing anticipatory from reactive metabolic regulation is interesting. The translational framing helps place the work in a broader context of sleep, circadian biology, and metabolic health.

Weaknesses:

At the same time, the evidence supporting the conclusions is somewhat limited. The metabolomics data were not newly generated but repurposed from prior work, reducing novelty. The biological replication in the respirometry assays is low, with only a small number of chambers per genotype. Importantly, respiratory parameters in flies are strongly influenced by locomotor activity, yet no direct measurements of activity were included, making it difficult to separate intrinsic metabolic changes from behavioral differences in mutants. In addition, repeated claims of "mitochondrial stress" are not directly substantiated by assays of mitochondrial function. The study also excluded female flies entirely, despite well-documented sex differences in metabolism, which narrows the generality of the findings.

# Author comment: Author response

**DOI:** 10.7554/eLife.108681.3.sa4

**Authors:** Farheen Akhtar, Dania M Malik, Arjun Sengupta, Paula Haynes, Andrew D Nguyen, C Jaco Klok, Amita Sehgal, Aalim Weljie

**Affiliations:** University of Pennsylvania PHILADELPHIA United States; University of Pennsylvania PHILADELPHIA United States; University of Pennsylvania PHILADELPHIA United States; Massachusetts General Hospital Boston United States; University of Pennsylvanialvania Philadelphia United States; Arizona State University Henderson United States; University of Pennsylvania Philadelphia United States; University of Pennsylvania PHILADELPHIA United States

The following is the authors’ response to the original reviews.

**Public Reviews:**

**Reviewer #1 (Public review):**

Summary:

This study by Akhtar et al. aims to investigate the link between systemic metabolism and respiratory demands, and how sleep and the circadian clock regulate metabolic states and respiratory dynamics. The authors leverage genetic mutants that are defective in sleep and circadian behavior in combination with indirect respirometry and steady-state LC-MS-based metabolomics to address this question in the Drosophila model.

First, the authors performed respirometry (on groups of 25 flies) to measure oxygen consumption (VO2) and carbon dioxide production (VCO2) to calculate the respiratory quotient (RQ) across the 24-hour day (12h:12h light-dark cycle) and assess metabolic fuel utilization. They observed that among all the genotypes tested, wild type (WT) flies and per0 flies in LD and WT flies in DD exhibit RQ >1. They concluded the >1 RQ is consistent with active lipogenesis. In contrast, the short-sleep mutants fumin (fmn) and sleepless (sss) showed significantly different RQ; the fmn exhibits a slight reduction in RQ values, suggesting increased reliance on carbohydrate metabolism, while sss exhibits even lower RQ (0.94), consistent with a shift toward lipid and protein catabolism.

The authors then proceeded to bin these measurements in 12-hour partitions, ZT0-12 and ZT12-24, to assess diurnal differences in average values of VO2, VCO2, and RQ. They observed significant day-night differences in metabolic rates in WT-LD flies, with higher rates during the day. The diurnal differences remain in the short-sleep mutants, but the overall metabolic rates are higher. WT-DD flies exhibit the lowest respiratory activity, although the day-night differences remain in free-running conditions. Finally, per01 mutants exhibit no significant change in day-night respiratory rates, suggesting that a functional circadian clock is necessary for diurnal differences in metabolic rates.

They then performed finer-resolution 24-hour rhythmic analysis (RAIN and JTK) to determine if VO2, VCO2, and RQ exhibit 24-hour rhythmic and if there are genotypespecific differences. Based on their criteria, VCO2 is rhythmic in all conditions tested, while VO2 is rhythmic in all conditions except in fmn-LD. Finally, RQ is rhythmic in all 3 mutants but not in WT-LD and WT-DD. Peak phases for the rhythms were deduced using JTK lag values.

The authors proceeded to leverage a previously published steady-state metabolite dataset to investigate the potential association of RQ with metabolite profiles. Spearman correlation was performed to identify metabolites that exhibit coupling to respiratory output. Positive and negative lag analysis were subsequently performed to further characterize these associations based on the timing of the metabolite peak changes relative to RQ fluctuations. The authors suggest that a positive lag indicates that metabolite changes occur after shifts in RQ, and a negative lag signifies that metabolite changes precede RQ changes. To visualize metabolic pathways that exhibit these temporal relationships, a clustered heatmap and enrichment analysis were performed. Through these analyses, they concluded that both sleep and circadian systems are essential for aligning metabolic substrate selection with energy demands, and different metabolic pathways are mis regulated in the different mutants with sleep and circadian defects.

We thank the reviewer for summarizing the contributions made by this manuscript.

Strength:

The research questions this study explores are significant, given that metabolism and respiratory demand are central to animal biology. The experimental methods used, including the well-characterized fly genetic mutants, the newly developed method for indirect calorimetry measurements, and LC-MS-based metabolomics, are all appropriate. This study provides insights into the impact of sleep and circadian rhythm disruption on metabolism and respiratory demand and serves as a foundation for future mechanistic investigations.

We thank the reviewer for the positive comments.

Weaknesses:

There are some conceptual flaws that the authors need to address regarding circadian biology, and some of the conclusions can be better supported by additional analysis to provide a stronger foundation for future functional investigation.

At times, the methods, especially the statistical analysis, are not well articulated; they need to be better explained.

Thank you for this suggestion and have revised and we expanded the Methods and figure legends to improve transparency and reproducibility.

Specifically, we have:

(i) Strengthened the rhythmicity description by specifying that rhythmicity was assessed in Nitecap using the RAIN algorithm with FDR-adjusted p-values (significant p ≤ 0.05; trending 0.05–0.1), and that period and peak phase were estimated with JTK_CYCLE (JTK lag = peak phase), with per-genotype period, phase, and p-values reported in Table 1;

(ii) Clarified sample size and replication by adding these lines to the methods section and indicating sample sizes in figure legends. Figure legends now report n (chambers) and SEM.

“Each genotype measurement represents an average of ~300 flies (25 flies per chamber × 4 chambers per experiment × 3 experimental days). The chamber was treated as the experimental unit for all analyses.”

In addition, we have expanded the description of metabolomics-respirometry correlation analyses to include dataset structure, time-matching across ZT, normalization steps, the use of Spearman correlations, and interpretation of lagged associations.

(iii) We have added these lines to the methods section:

“To integrate respirometry with metabolomics, RQ was recorded continuously at 1-second resolution and averaged into 5-minute bins. Because steady-state metabolite measurements were acquired at 2-hour intervals, we extracted the RQ values corresponding to each 2-hour Zeitgeber Time (ZT) sampling point from the 5-minutebinned dataset to generate time-matched RQ-metabolite pairs. We additionally evaluated temporal relationships using a lag analysis by systematically shifting the RQ time series relative to the metabolite time points (−120, −60, −30, −15, −5, +5, +15, +30, +60, and +120 minutes). Metabolite abundances were normalized as described above, and associations between RQ and individual metabolites were quantified using Spearman rank correlations (ρ) at each lag. Metabolites showing strong associations (e.g., |ρ| > 0.7 with nominal p < 0.05) were carried forward for visualization and summary, and lag direction was interpreted as metabolites preceding (negative lag) or following (positive lag) changes in RQ.”

**Reviewer #2 (Public review):**

This is an innovative and technically strong study that integrates dual-gas respirometry with LC-MS metabolomics to examine how sleep and circadian disruption shape metabolism in Drosophila. The combination of continuous O_2_/CO_2_ measurements with high-temporal-resolution metabolite profiling is novel and provides fresh insight into how wild-type flies maintain anticipatory fuel alignment, while mutants shift to reactive or misaligned metabolism. The use of lag-shift correlation analysis is particularly clever, as it highlights temporal coordination rather than static associations. Together, the findings advance our understanding of how circadian clocks and sleep contribute to metabolic efficiency and redox balance.

We thank the reviewer for the positive comments.

However, there are several areas where the manuscript could be strengthened.

The authors should acknowledge that their findings may be gene specific. Because sleep deprivation was not performed, it remains uncertain whether the observed metabolic shifts generalize to sleep loss broadly or are restricted to the fmn and sss mutants. This concern also connects to the finding of metabolic misalignment under constant darkness despite an intact clock.

We agree that our findings should be framed as genotype- and condition-specific. The phenotypes we report arise from chronic, genetically encoded sleep loss (fmn, sss) and clock loss (per01); because acute sleep deprivation was not performed, we do not claim these effects generalize to sleep loss broadly. This also bears on the reviewer's point about constant darkness: the metabolic misalignment we observe in WT-DD occurs despite an intact clock, and we therefore interpret it as a consequence of removing external light-dark cues under our conditions. We have scoped the claims accordingly in the Abstract, Results, and Discussion (subsection “Metabolic Desynchrony and Redox Imbalance in Wild-Type Flies Under Constant Darkness, DD”).

The text now reads as follows:

“We restrict our conclusions to the genotypes and conditions tested (*fmn*, *sss*, and *per^01^*), and we do not generalize these effects to acute sleep deprivation because sleep deprivation was not performed in this study. Accordingly, the ‘metabolic misalignment’ observed in constant darkness (DD) likely results from a decrease in synchrony due to the removal of external light:dark cues.”

The conclusion that external entrainment is essential for maintaining energy homeostasis in flies may not translate to mammals. It would help to reference supporting data for the finding and discuss differences across species. Ideally, complementary circadian (lightdark cycle disruption) or sleep deprivation (for several hours) experiments, or citation of comparable studies, would strengthen the generality of the findings.

Thank you. We have tempered the interpretation and expanded both the discussion and its citations. We now (i) avoid stating that external entrainment is universally “essential” for energy homeostasis, (ii) explicitly discuss fly-mammal differences (sleep architecture, thermoregulation, feeding control, and entrainment mechanisms), and (iii) anchor the translational comparison to the mammalian circadian-misalignment and sleep-loss literature already integrated in our Discussion (refs [3, 43-46]), noting that establishing cross-species generality will require additional paradigms (constant light, acute sleep deprivation).

The text now reads as follows:

“These phenotypes parallel mammalian systems, where sleep loss and circadian misalignment are linked to elevated basal metabolic rate, a shift toward carbohydrate oxidation and lipid/protein catabolism, and blunted, phase-shifted respiratory oscillations [3, 43-46]; physiological differences between flies and mammals nonetheless caution against direct mechanistic extrapolation. In constant darkness, our DD data show that endogenous free-running regulation persists but that removing external light-dark cues degrades temporal coordination between respiration and metabolism; however, we acknowledge that the coupling may be different in mammals.”

Figures 1-4 are straightforward and clear, but when the manuscript transitions to the metabolite-respiration correlations, there is little description of the metabolomics methods or datasets, which should be clarified.

Thank you for noting this. We agree that the transition to the metabolite–respiration correlation analyses required clearer description of the metabolomics datasets and processing. We have revised the Methods and the corresponding Results text to briefly summarize the metabolomics dataset parameters and workflow, including how metabolomics and respirometry measurements were time-matched across ZT, the normalization procedures applied prior to analysis, the use of Spearman rank correlations, and how we interpret lagged relationships between metabolite abundance and respiratory outputs.

The text now reads as follows:

Methods:

“Metabolomics-respirometry integration and lag analysis

To integrate respirometry with metabolomics, RQ was recorded continuously at 1-second resolution and averaged into 5-minute bins. Because steady-state metabolite measurements were acquired at 2-hour intervals, we extracted the RQ values corresponding to each 2-hour Zeitgeber Time (ZT) sampling point from the 5-minutebinned dataset to generate time-matched RQ-metabolite pairs. We additionally evaluated temporal relationships using a lag analysis by systematically shifting the RQ time series relative to the metabolite timepoints (−120, −60, −30, −15, −5, +5, +15, +30, +60, and +120 minutes). Metabolite abundances were normalized as described above, and associations between RQ and individual metabolites were quantified using Spearman rank correlations (ρ) at each lag. Metabolites showing strong associations (e.g., |ρ| > 0.7 with nominal p < 0.05) were carried forward for visualization and summary, and lag direction was interpreted as metabolites preceding (negative lag) or following (positive lag) changes in RQ.”

Results:

“Temporal Profiling of Respiratory Quotient in Wild-Type Flies Under Light-Dark Conditions

RQ values corresponding to each 2-hour Zeitgeber Time (ZT) point were extracted from the 5-minute-binned dataset. Building on this alignment, we explored temporal relationships by systematically shifting the RQ time series by −120, −60, −30, −15, −5, +5, +15, +30, +60, and +120 minutes relative to the metabolite dataset. The continuous respirometry time series showed an oscillatory day-night pattern in RQ; metabolomics was then used to relate time-matched and lagged metabolite dynamics to RQ patterns (Figure 4).”

The Discussion is at times repetitive and could be tightened, with the main message (i.e., wild-type flies align metabolism in advance, while mutants do not) kept front and center.

Thank you for this helpful suggestion. We have revised the Discussion to reduce repetition and improve focus by keeping the central takeaway explicit throughout, and by consolidating overlapping paragraphs into a more streamlined narrative.

We added this revision at the start of the Discussion, in the opening subsection “Temporal Misalignment Alters Fuel Utilization and Respiratory Rhythms.”

The Discussion now reads as follows:

“Across the manuscript, the central takeaway is that wild-type flies under LD exhibit anticipatory alignment of fuel selection with time of day, whereas short-sleep mutants (fmn, sss) and clock-disrupted flies (per01) show reactive or misaligned metabolism under our conditions. We therefore focus the Discussion on loss of temporal coordination between respiratory output and pathway-level metabolism, rather than reiterating rate changes alone.”

Terms such as "anticipatory" and "reactive" should be defined early and used consistently throughout.

Thank you for this suggestion. We agree and have revised the manuscript to define these terms early (at first use) and apply them consistently throughout. We added this definition in two places:

(i) In the Results, at the start of the metabolomics-respirometry integration section where we first introduce the lag analysis, and

(ii) In the Methods, within the paragraph describing the lag analysis workflow, using identical wording.

The text now reads as follows:

“We define ‘anticipatory’ as metabolite changes that precede the associated respiratory shift (negative lag) and ‘reactive’ as changes that follow or coincide with the respiratory shift (positive lag), and we use these terms consistently throughout.”

Overall, this is a strong and novel contribution. With clarification of scope, refinement of presentation, and a more focused Discussion, the paper will make a significant impact.

We again thank the reviewer for the positive comments.

**Reviewer #3 (Public review):**

Summary:

The authors investigate how sleep loss and circadian disruption affect whole-organism metabolism in *Drosophila melanogaster*. They used chamber-based flow-through respirometry to measure oxygen consumption and carbon dioxide production in wild-type flies and in mutants with impaired sleep or circadian function. These measurements were then integrated with a previously published metabolomics dataset to explore how respiratory dynamics align with metabolic pathways. The central claim is that wild-type flies display anticipatory coordination of metabolic processes with circadian time, while mutants exhibit reactive shifts in substrate use, redox imbalance, and signs of mitochondrial stress.

We thank the reviewer for summarizing the contributions made by this manuscript.

Strengths:

The study has several strengths. Continuous high-resolution respirometry in flies is challenging, and its application across multiple genotypes provides good comparative insight. The conceptual framework distinguishing anticipatory from reactive metabolic regulation is interesting. The translational framing helps place the work in a broader context of sleep, circadian biology, and metabolic health.

We thank the reviewer for the positive comments.

Weaknesses:

At the same time, the evidence supporting the conclusions is somewhat limited. The metabolomics data were not newly generated but repurposed from prior work, reducing novelty.

Thank you for raising this point. We now make the provenance of the metabolomics dataset explicit in the manuscript. Importantly, the current study uses this dataset in a new analytical context by integrating it with continuous VCO_2_/VO_2_ respirometry through timematched and lag-aware analyses. This approach allows us to evaluate dynamic relationships between respiratory output and metabolite profiles across circadian time, which was not addressed in the original metabolomics study. We have clarified this point in the Introduction and Methods.

The text now reads as follows:

In the Introduction:

“To provide a more comprehensive perspective on metabolic regulation, we complemented newly generated respiratory measurements with steady-state metabolomic profiling using liquid chromatography-mass spectrometry (LC-MS) data previously published from our group [27]. This integrative framework enabled timematched and lag-aware analysis of respiratory output and metabolite profiles across Zeitgeber time in the LD cycle….”

In the Methods:

“The metabolomics dataset analyzed in this study was previously published and is publicly available, as described in detail in [27, 31]. In the present study, these data were integrated with respirometry measurements to assess temporal relationships between metabolite abundance and respiratory output.”

The biological replication in the respirometry assays is low, with only a small number of chambers per genotype.

Thank you for highlighting this concern. We suggest that this is a lack of clarity in our initial description of the design and that the replication structure should be stated more explicitly. We have revised the Methods and all relevant figure legends to clearly report biological replication using the chamber as the experimental unit, including n (number of chambers) per genotype and the associated error structure. We also clarify sampling depth by stating that each genotype measurement reflects an average of ~300 flies (25 flies per chamber × 4 chambers per experiment × 3 experimental days). This information is now reported consistently to make the unit of analysis transparent.

We added this clarification in the Methods under “Respirometry Setup” (where chamber loading and experimental design are described) and ensured that each relevant figure legend explicitly reports n (chambers) and SEM.

The text now reads as follows:

“Each genotype measurement represents an average of ~300 flies (25 flies/chamber × 4 chambers/experiment × 3 experimental days), with the chamber as the experimental unit; n (chambers) and SEM are reported in each figure legend.”

Importantly, respiratory parameters in flies are strongly influenced by locomotor activity, yet no direct measurements of activity were included, making it difficult to separate intrinsic metabolic changes from behavioral differences in mutants.

A detailed timing comparison between behavior (feeding and locomotion) compared to respirometry is given in our master response to Reviewer 1, Major comment 4.

In addition, repeated claims of "mitochondrial stress" are not directly substantiated by assays of mitochondrial function.

Thank you. We agree that “mitochondrial stress” requires direct functional evidence. We therefore directly assayed mitochondrial respiration (baseline gut-tissue OCR in fmn and per01 versus iso31 controls), added a new Methods subsection and Figure 9, and reframed our wording from “mitochondrial stress/impairment” to altered (elevated) baseline mitochondrial respiration. The experimental details are now in the Methods and the result in the Results (both quoted below). sss was not assayed, so we removed the functional mitochondrial-stress claim for sss; we retain Vaccaro et al. (2020) as prior support for fmn.

Methods — new subsection “Gut Tissue Respirometry” now reads: “Oxygen consumption rate (OCR) was measured in dissected gut tissue from iso31, fmn, and per01 flies using the Resipher System (Lucid Scientific, GA, USA). Baseline OCR (fmol/mm^2^/s) was averaged over a 24-hour window following a 12-hour acclimation; values from 3 independent runs were pooled, median-normalized to iso31 within each run, and log2(x+10)-transformed. A single iso31 outlier (third per01 run) was excluded; no other values were removed. Each mutant was compared with iso31 using the MannWhitney test (GraphPad Prism 10), with significance at p < 0.05 (fmn vs iso31, n = 12 vs 12; per01 vs iso31, n = 12 vs 11 after excluding one iso31 outlier).”

The text has been added to Results:

“Because pathway-level metabolomics implicated mitochondrial pathways in the sleep and circadian mutants, we directly assayed mitochondrial respiration by measuring baseline oxygen consumption rate (OCR) in dissected gut tissue from fmn and per01 relative to iso31 controls. Both fmn and per01 guts showed significantly elevated baseline OCR (fmn vs iso31, n = 12 vs 12; per01 vs iso31, n = 12 vs 11 across 3 runs; Mann-Whitney test, p<0.05; Figure 9A,B). Because only baseline OCR was measured, we interpret this as altered (elevated) baseline mitochondrial respiration rather than reduced capacity or a specific coupling defect (Figure 9).”

Discussion- *fmn*:

“These interpretations are further supported by gut-tissue respirometry showing elevated baseline mitochondrial respiration in fmn relative to iso31 controls. Together with prior evidence of ROS accumulation (oxidative stress) in fmn (Vaccaro et al., Cell, 2020), these functional data indicate that chronic sleep loss in fmn is associated with altered mitochondrial respiration.”

Discussion- *per01*:

“Gut-tissue respirometry in per01 likewise showed elevated baseline mitochondrial respiration, providing functional evidence consistent with the metabolomic signatures of disrupted redox balance and mitochondrial metabolism.”

The study also excluded female flies entirely, despite well-documented sex differences in metabolism, which narrows the generality of the findings.

Thank you for raising this point. We agree that sex is an important biological variable in metabolic regulation. While our Methods state that male flies were collected, we have now made this explicit and unambiguous by stating that only males were used for respirometry and metabolomics integration, and we have added this as a limitation in the Discussion, noting that sex-specific physiology could influence the magnitude and/or timing of the effects we report. We also highlight inclusion of females as an important future direction.

The text now reads as follows (Methods):

“Only male flies were used for all respirometry experiments and for integration with the metabolomics dataset. This was done to reduce variability introduced by female reproductive status (e.g., mating/egg production) and associated metabolic differences, enabling a clearer comparison across genotypes and lighting conditions.”

The text now reads as follows (Discussion):

“Because only males were analyzed, our conclusions may not generalize to females, which can show sex-specific metabolic physiology. Inclusion of female flies and direct sex comparisons across LD and DD conditions will be an important future direction. More specifically, females carry a higher reproductive and biosynthetic load (egg production) that typically raises metabolic rate and can shift RQ toward lipogenesis and alter the amplitude and phase of diurnal respiratory rhythms; females might therefore show larger or differently-timed effects than the males studied here.”

**Recommendations for the authors:**

**Reviewer #1 (Recommendations for the authors):**

Major comments:

(1) The authors appear to be using WT-DD as a condition to disrupt circadian rhythm (line 216). Although rhythmicity is often dampened in DD compared to in LD, circadian rhythm is defined as rhythm in a constant condition (e.g., DD) after entrainment. WT-DD is not a condition that the authors should use if they want to disrupt circadian rhythms in flies. WTLL would be a better condition to use since flies become arrhythmic in LL, not DD.

Thank you for this clarification. We agree that DD is not a circadian-disrupting condition; circadian rhythmicity is defined by rhythms that persist under constant conditions after entrainment. Our intent was not to treat WT-DD as arrhythmic, but to use DD to assess free-running circadian regulation in the absence of external light–dark cues. We have revised the manuscript to clearly distinguish diurnal rhythms under LD from free-running circadian rhythms under DD, and to avoid implying that DD abolishes rhythmicity.

The Results text now reads as follows:

“We used constant darkness (DD) to assess free-running circadian regulation in the absence of external light–dark cues.”

In the Discussion, in the subsection “Metabolic Desynchrony and Redox Imbalance in Wild-Type Flies Under Constant Darkness,” we revised the interpretation of WT-DD to clarify that the observed metabolic effects reflect removal of external light–dark cues under our experimental conditions, while avoiding overgeneralization.

The Discussion text now reads as follows:

Our DD data show that endogenous free-running circadian regulation persists but that removing external light–dark cues degrades the temporal coordination between respiration and metabolism, indicating that external entrainment normally strengthens this coordination under our conditions; however, we acknowledge that the coupling may be different in mammals.

(2) The authors need to be more precise in the use of the term "circadian" throughout the manuscript. When describing 24h rhythmicity in the LD condition in flies, they can use the diurnal rhythm. Circadian rhythm is an endogenous rhythm without external time cues (e.g., DD rhythm).

Thank you for this point. We agree and have revised the manuscript to use terminology consistently: rhythms measured under LD are now referred to as diurnal (LD) rhythms/patterns, and the term circadian is reserved for endogenous free-running rhythms in DD. We updated the Methods, Results, Discussion and figure legends throughout to correct instances where LD rhythmicity was previously labeled as “circadian.”

We added this clarification in the Methods (“*Drosophila* Strains, Entrainment and Collection”) and in the Results/figure legends where LD time courses are described.

The text now reads as follows (Methods):

“Male flies were collected shortly after eclosion and entrained in light-dark (LD) incubators for a minimum of three days before diurnal (LD) time-course collection across Zeitgeber time (ZT).”

We revised the Results text where WT-DD and LD time courses are described, replacing imprecise references to ‘circadian disruption’ or ‘circadian cycle’ with ‘free-running conditions in DD,’ ‘24-hour cycle,’ or ‘diurnal pattern under LD,’ as appropriate. We also revised the Discussion to avoid describing LD patterns as circadian and to avoid implying that DD disrupts circadian rhythmicity.

(3) Lines 253-256: The authors' interpretation of this data is not accurate. The authors observed significant day-night differences in VO2 and VCO2 in WT-DD. This suggests there is circadian control over metabolism rhythms. The authors noted there is "limited circadian control".

Thank you for pointing this out. We agree that the significant day-night differences in VO_2_ and VCO_2_ in WT-DD support persistent endogenous (circadian) control of respiratory rhythms under constant darkness. We have revised the Results text (lines 253-256) to remove the statement implying “limited circadian control” and instead describe the WTDD effect as maintained rhythmicity with altered amplitude and/or phase relative to LD, rather than loss of rhythmic regulation.

We added this revision in the Results section under “Diurnal Variation in CO_2_ Production, O_2_ Consumption, and Respiratory Quotient Across Genotypes” (WT-DD description; lines 253-256).

The text now reads as follows:

WT-DD flies, maintained in constant darkness, exhibited the lowest overall respiratory activity. Despite the absence of environmental light cues, VCO_2_ and VO_2_ retained significant day-night differences (Figure S4), consistent with persistent free-running circadian control in constant darkness (with altered amplitude and/or phase relative to LD).

(4) Besides sleep, metabolic rates are known to be affected by food consumption. Measuring food consumption of the sleep and circadian mutants might provide insights into whether the metabolic rates are more affected by changes in sleep profile or food consumption. This might also be important given fumin displays impaired dopamine transport function and defective dopamine reuptake, and dopamine is known to affect eating behavior. This was not considered and/or discussed.

It is technically challenging to measure feeding during respirometry, so we acknowledge it as a limitation. To test whether behavioral timing could explain our results, we compared our respiratory rhythms with the feeding and activity rhythms reported in Malik et al. (2026); the comparison and its interpretation are now in the Discussion (quoted below). This is our master response to the activity/feeding concern and is crossreferenced from Reviewer 3’s public review and Recommendation 1; feeding and activity were not measured for sss.

We added this clarification in the Discussion (Limitations/confounds) where we address potential behavioral contributors (activity/feeding) to respirometry outcomes.

The text now reads as follows:

“Locomotor activity and feeding could not be measured during the respirometry recordings, so genotype differences in respiratory parameters should be interpreted with caution. To assess whether behavioral timing could account for these differences, we compared our respiratory rhythms with the feeding and activity rhythms reported for these genotypes in Malik et al. (2026): wild-type feeding peaked at ZT ~3.25 and fmn feeding was phase-delayed to ZT ~4.5, while fmn also showed elevated locomotor activity, particularly during the dark period. In our data the fmn VCO2 rhythm peaks at ZT ~3.25 (RQ at ZT ~4.25), so the respiratory peak slightly precedes the feeding peak; the phase of the fmn respiratory rhythm is therefore not driven by feeding, although the elevated activity of fmn may contribute to its higher overall metabolic rate. Feeding and activity were not measured for sss.”

(5) It is not clear whether the authors are simply analyzing the SAME dataset in Figure 1, Figure S4, and Figure 2-3, but just with different resolutions. They need to better articulate this point.

We thank the reviewer for pointing this out. While the source data for Figures 1, 2-3, and Figure S4 use the same underlying respirometry dataset, they present different analyses to address specific questions. Figure 1 shows the full time-course traces (fine time bins), Figures 2-3 extract rhythmicity metrics (e.g., period/phase) from those same time-series, and Figure S4 collapses the same data into simple day (ZT0-12) vs night (ZT12-24) averages.

We added this clarification in the Figure legends for Figure 1, Figures 2-3, and Figure S4, and also noted it in the Methods where the respirometry analysis outputs (time-series binning, rhythmicity analysis, and day/night averaging) are described.

(6) Figure 3 and page 14: What are their criteria for differentiating "conserved" vs "distinct" phase alignment? It is not clear whether this conclusion is supported by any statistical analysis.

We now specify that the “conserved” vs “distinct” phase descriptions refer to early- vs late-peaking rhythms, and we state the criterion explicitly: a phase was called “conserved” when it fell within ±3 h of the WT-LD peak. The revised text reads: “VO_2_ peaked … suggesting conserved phase alignment, with all phases within ±3 h of WT-LD (Figure 3, Table 1)”; “RQ peaked … indicating distinct phase alignment, with the sleep-mutant phases ~8 h apart (Figure 3, Table 1).”

The text now reads as follows:

“VO_2_ peaked …suggesting conserved phase alignment, with all phases within ±3 h of WT-LD (Figure 3, Table 1)”

“RQ peaked … indicating distinct phase alignment compared to respiratory output, with phases of the sleep mutants 8 h apart (Figure 3, Table 1).”

(7) Figure 4: The authors need to provide more details as to how the metabolite dataset was utilized to generate this figure and how they made the conclusion that their analysis "revealed a distinct circadian rhythmicity in RQ, characterized by oscillatory patterns indicative of coordinated substrate utilization across the day-night cycle".

We have clarified how the respirometry and metabolomics data are used for Figure 4 and corrected the overstated rhythmicity claim:

(1) The RQ patterning in Figure 4 is derived from the continuous respirometry time series, not from the metabolomics dataset, which is used only for the time-matched and lagged correlation analyses that relate metabolite dynamics to RQ. (2) We removed the statement that the analysis “revealed a distinct circadian rhythmicity in RQ”: RQ was not statistically rhythmic in WT-LD or WT-DD (Table 1), and Figure 4 instead shows the day-night RQ pattern that serves as the reference for the lag-based metabolite correlations. We revised the Methods, the Results paragraph introducing Figure 4, and the Figure 4 legend accordingly.

The text now reads as follows:

“Respiratory quotient (RQ) was recorded continuously and averaged into 5-minute bins. To integrate with metabolomics collected every 2 hours, we extracted the corresponding 2-hour ZT RQ values and performed a lag analysis (−120 to +120 min) to relate metabolite dynamics to RQ patterns; rhythmicity of RQ itself was assessed from the respirometry time series.”

(8) It is unclear why the examples of hydroxyhexadecenoylcarnitine and quinolinate were chosen to be presented in Figure 5a. The authors should clarify their choice of these two examples. Also, the authors should generate a supplemental table with the "several metabolites demonstrating strong correlations (line 294).

Thank you for this suggestion. Hydroxyhexadecenoylcarnitine and quinolinate were selected as representative examples, and we agree that the metabolites supporting the strong RQ-associated correlations should be provided more explicitly. We have now added a Supplementary Table listing the metabolites demonstrating strong correlations with RQ across WT-LD, *fmn*, *sss*, *per^01^*, and WT-DD conditions, using the same selection criterion applied in the heatmap analyses (|ρ| ≥ 0.7, p < 0.05).

We also revised the Results text near the statement describing strong metabolite-RQ correlations to direct readers to this new table.

The text now reads as follows:

Hydroxyhexadecenoylcarnitine and quinolinate are among the strongest positively- and negatively-lagged RQ-correlated metabolites in WT-LD (ρ = +0.78 at +120 min and ρ = −0.77 at −120 min; Supplementary Table 1), illustrating the two opposite lag directions of the workflow. The full set of metabolites showing strong RQ-associated correlations across WT-LD, *fmn*, *sss*, *per^01^*, and WT-DD conditions is provided in Supplementary Table 1.

(9) Although Spearman correlation analysis suggests some correlation between RQ and the two metabolites shown in Figure 5, the correlation shown in Figure 5b does not appear to be compelling. Results shown in Figure 5b do not provide confidence that conclusions based on clustered heatmap analysis shown in Figures 6 to 8 are meaningful. In addition to Spearman correlation, the authors might consider performing additional statistical methods to provide further support.

Thank you for this comment. We suggest that Fig. 5b was not explained clearly and have clarified. Figure 5b is a lag analysis, not a separate correlation result: it shows how the Spearman correlation changes when the RQ time series is shifted forward or backward in time relative to the metabolite timepoints. The goal is to illustrate lead-lag timing (which shift gives the strongest association), rather than to present a single “strong” correlation as standalone proof.

To address the concern about confidence in the heatmap-based results (Figs. 6-8), we have strengthened the reporting by providing effect sizes (Spearman ρ) and lag for the metabolite-respirometry associations (now included as Supplementary Table 1). This allows readers to evaluate the statistical support underlying the clustering, beyond the visual patterns in the heatmaps.

We additionally report multiple-testing–corrected significance for the metabolite–RQ correlations (Benjamini–Hochberg FDR) alongside nominal p in Supplementary Table 1, using the same correction already applied to the pathway enrichment in Supplementary Table 2.

Minor comments:

(1) Line 82: The authors should clarify what they mean by "circadian collection". Except for WT-DD, my interpretation is that they collected their samples in LD, so that would not be "circadian collection".

Thank you for catching this. We agree that “circadian collection” was imprecise. We have revised line 82 to clarify that samples collected under LD were collected across diurnal (LD) time (ZT), and we now reserve “circadian” specifically for collections under constant conditions (DD). We also updated the wording throughout the manuscript to maintain this distinction consistently. We added this clarification in the Methods section “*Drosophila* Strains, Entrainment and Collection” (line 82).

The text now reads as follows:

“Male flies were collected shortly after eclosion and entrained in light-dark (LD) incubators for a minimum of three days before diurnal (LD) time-course collection across Zeitgeber time (ZT).

(2) The authors cited Frayn 1983 to indicate how the RQ value can be used to reflect metabolic fuel utilization. Is this interpretation accepted for all animals, including flies?

RQ is widely used in indirect calorimetry as an index of relative substrate utilization, including in small model organisms, but we agree that it should be interpreted with appropriate caveats. We have revised the manuscript to clarify that we interpret RQ conservatively as reflecting relative shifts in substrate utilization over time and between genotypes, rather than as a precise quantitative measure of absolute carbohydrate versus lipid oxidation.

We made this change in two places: in the Introduction, where RQ is introduced and Frayn is cited, and in the Methods, under “Carbon Dioxide and Oxygen Analysis and Calculations,” where RQ is defined.

The text now reads as follows:

Introduction: “These measurements allow for the estimation of energy expenditure and respiratory quotient (RQ), which can provide an index of relative substrate utilization, with appropriate caveats[13, 14]. In this study, we interpret RQ conservatively as reflecting relative shifts in substrate utilization over time and between genotypes, rather than as a precise quantitative measure of absolute carbohydrate versus lipid oxidation.”

Methods: “RQ was used as an index of relative shifts in substrate utilization over time and between genotypes, interpreted conservatively rather than as a precise measure of absolute carbohydrate versus lipid oxidation as this has not been directly characterized in flies.

(3) Figure S4: Since the authors are comparing day-night differences, they should plot them in the same graph to make it easier to compare.

We have revised Figure S4 to plot day and night within the same graph/panel for each metric (VCO_2_, VO_2_, RQ), using side-by-side day vs night groupings per genotype to facilitate direct visual comparison, and we updated the legend accordingly.

(4) Line 252: When comparing diurnal differences, the authors should not use the word "rhythm". Pattern or profile might be a better word to use.

We appreciate the suggestion. Where we use “rhythm” we refer specifically to 24-hour oscillations established statistically by JTK_CYCLE and RAIN (Figures 2-3, Table 1); for the coarser day-versus-night comparisons we agree “pattern” or “profile” is preferable and have adopted it there. We have gone through the manuscript to apply this distinction consistently.

(5) Figure 6a: larger font labels are necessary for the metabolites.

Thank you. We have revised Figure 6a to increase the metabolite label font size for improved readability.

**Reviewer #3 (Recommendations for the authors):**

(1) Activity controls: To strengthen the paper, include or reference direct measures of locomotor activity (e.g., DAM system). This would allow the separation of metabolic changes from behavioral differences and would enable better analysis of circadian patterns.

This activity/feeding confound is addressed in full in our response to Reviewer 1, Major comment 4; we cross-reference it here to avoid repetition.

(2) Mitochondrial function: "mitochondrial stress" should be supported by additional assays such as mitochondrial enzyme activities, high-resolution respirometry, or reactive oxygen species measurements.

See our full response to the mitochondrial point in Reviewer #3's public review above, including new Figure 9 and the Gut Tissue Respirometry Methods.

(3) Sex differences: Provide a clear justification for excluding female flies. If feasible, incorporate female data or explicitly discuss how sex differences could alter metabolic outcomes.

This is addressed in the public review for reviewer 3.

(4) Statistical presentation: Increase n. Clarify in figure legends whether error bars represent SD or SEM and ensure consistency across all figures (Figure legends).

We have revised all figure legends to clearly state that error bars represent SEM and ensured this is applied consistently across all figures. We also explicitly report the n for each genotype (with chambers as the experimental unit) in the relevant legends.

The text now reads as follows:

“Error bars represent SEM, and n denotes the number of chambers (experimental units) per genotype.”

(5) Sample size and replication: Indicate more clearly that the chamber, not the individual fly, is the experimental unit. Discuss limitations of replication and statistical power in the text.

Thank you for this comment. We have clarified throughout the Methods and figure legends that the chamber (not the individual fly) is the experimental unit. We now state that each genotype measurement reflects an average of 300 flies (25 flies/chamber × 4 chambers/experiment × 3 experimental days), and we report n (number of chambers) and the error structure (SEM) for each genotype.

Added to Discussion:

“We also acknowledge the limits of this replication: with n = 12 chambers per genotype, statistical power to detect small-magnitude differences and subtle phase shifts is limited, and negative calls (e.g., arrhythmicity) should be interpreted with this caveat.”

(6) Writing and clarity: (a) Streamline the Discussion to focus on mechanistic themes (anticipatory vs reactive alignment, substrate shifts, redox imbalance).

(b) The opening phrase ("Precise temporal regulation of metabolism by sleep and circadian rhythms is essential for dynamic energy homeostasis") is dense, vague, and difficult to interpret. Consider rephrasing to something more concrete, for example: "Sleep and circadian rhythms tightly control when and how the body uses energy, but we do not yet know exactly how this timing connects to oxygen use and breathing needs."

Thank you for these helpful suggestions. We have revised the Discussion to reduce repetition and improve focus by organizing it around the key mechanistic themes raised by our data, including anticipatory vs reactive metabolic alignment, substrate-use shifts, and redox/mitochondrial imbalance. We revised the opening sentence of the Abstract to make the biological question more concrete and accessible, following the reviewer’s suggestion.
